# A High-Efficiency Data Collection Method Based on Maximum Recharging Benefit in Sensor Networks

**DOI:** 10.3390/s18092887

**Published:** 2018-08-31

**Authors:** Chao Sha, Qi-Wei Wang, Lu Zhang, Ru-Chuan Wang

**Affiliations:** 1 School of Computer Science, Software and Cyberspace Security, Nanjing University of Posts and Telecommunications, Nanjing 210003, China; wangqwedu@163.com (Q.-W.W.); zhanglucst@gmail.com (L.Z.); wangrc@njupt.edu.cn (R.-C.W.); 2 Jiangsu High Technology Research Key Laboratory for Wireless Sensor Networks, Nanjing 210003, China

**Keywords:** wireless sensor networks, data collection, balance of energy consumption, wireless recharging, Maximum Recharging Benefit

## Abstract

To reduce time delays during data collection and prolong the network lifetime in Wireless Rechargeable Sensor Networks (WRSNs), a type of high-efficiency data collection method based on Maximum Recharging Benefit (DCMRB) is proposed in this paper. According to the minimum number of the Mobile Data Collectors (MDCs), the network is firstly divided into several regions with the help of the Virtual Scan Line (VSL). Then, the MDCs and the Wireless Charging Vehicles (WCVs) are employed in each region for high efficient data collection and energy replenishment. In order to ensure the integrity of data collection and reduce the rate of packet loss, a speed adjustment scheme for MDC is also proposed. In addition, by calculating the adaptive threshold of the recharging request, those nodes with different energy consumption rates are recharged in a timely way that avoids their premature death. Finally, the limited battery capacity of WCVs and their energy consumption while moving are also taken into account, and an adaptive recharging scheme based on maximum benefit is proposed. Experimental results show that the energy consumption is effectively balanced in DCMRB. Furthermore, this can not only enhance the efficiency of data collection, but also prolong the network lifetime compared with the Energy Starvation Avoidance Online Charging scheme (ESAOC), Greedy Mobile Scheme based on Maximum Recharging Benefit (GMS-MRB) and First-Come First-Served (FCFS) methods.

## 1. Introduction

In recent years, Wireless Sensor Networks (WSNs for short) with sensing, computing and communication ability have gradually become essential components of the Internet of Things. Sensor nodes are often deployed in the wild with limited battery capacity and are difficult to replace. Therefore, how to balance the energy consumption of the whole network as far as possible, and meanwhile provide the nodes with continuous energy supply at the lowest cost is becoming more and more important for WSNs.

With the deepening of research, more and more energy efficient strategies are adopted to reduce the energy consumption of both sensors and networks. For example, we could optimize the deployment of nodes to balance their traffic load [1], adopt efficient communication protocols for data collection [2] or adjust the sampling frequency to reduce sending redundant data [3]. In addition, some studies are dedicated to low-power hardware design, low-complexity software implementation, and power-efficient wireless communication [4,5,6]. However, it is undeniable that nodes near the sink, for example, cluster heads, are more likely to die due to their large amount of data collecting and forwarding activity. This results in network disconnection and causes the “hotspot problem”. As important WSN nodes, sinks are always assumed to be static and placed in the center of the network. They usually act as a gateway between the sensor nodes and the end-users [7]. To solve this problem, Xie et al. [6] have analyzed the relationship between expected energy expenditure, packet loss ratio, end to end delay as well as WSN lifetime. They have then proposed a Residual Energy Aware scheme with adjustable Duty Cycle (READC) based on the fact that energy consumption is higher in the region near sinks, while it is lower in the areas far away from the sinks. 

Mobile Data Collectors (MDCs) have been frequently used in data gathering systems nowadays [8,9,10]. Generally, MDCs are resource-rich devices with more energy, higher communication power, and more powerful sensing as well as computing capabilities. Since the routing task has been partially or fully taken over by one or more MDCs, this approach can effectively eliminate the non-uniformity of energy consumption among sensor nodes and reduce their heavy traffic load.

Although the network connectivity and the energy efficiency could be improved with the help of MDCs, this cannot ensure the perpetual operation of the network. On the other hand, many studies have shown that energy harvesting from natural sources, such as solar, wind, thermal and vibration can effectively improve network performance and prolong network lifetimes. However, the effect of energy harvesting mainly depends on the environment. For example, in a solar harvesting system, the amount of harvested energy is determined by the time and strength of solar radiation. Thus, energy harvesting from the environment is not entirely applicable to WSNs [11]. In recent years, with the rapid development of energy harvesting and wireless recharging technologies, the advantages of Wireless Rechargeable Sensor Network (WRSN) become more and more obvious in extending WSNs’ lifetime and improving system robustness [12,13,14,15,16]. In a realistic scenario, a Wireless Charging Vehicle (WCV) travels across the network and charges all the rechargeable sensor nodes [17]. The recharge sequences are often calculated out in advance such that nodes can be recharged before energy depletion [18]. Each node would be charged only once by WCV in one charging cycle [17]. After visiting all the nodes, the WCV moves back to the service station. Ideally, the lifetime of a WRSN can be extended to infinitely long for perpetual operations [18]. However, the main drawback of energy harvesting is the low efficiency of recharging, since the power output of energy harvesting devices is relatively low compared to the energy consumption on sensing and communications. To solve it, one or more mobile Wireless Charging Vehicles (WCVs) with a certain amount of energy are commonly used in WRSNs to recharge the sensor nodes. For example, a proof-of-concept prototype of WRSN has been established by Peng et al. [19], and experiments have been conducted to evaluate its feasibility as well as the energy replenishment performance in small-scale WSN. 

Both MDCs and WCVs, however, expend significant time and energy for moving, and most studies do not consider the cooperativity between them. Therefore, how to determine the optimal number of MDCs and WCVs and how to optimize the moving path to ensure real-time data collection as well as high efficient wireless recharging, has become a hot issue to be solved urgently in WRSNs.

The remainder of this paper is organized as follows: related works and the network model are described in Section 2 and Section 3, respectively. In Section 4, we propose a type of multi-MDCs based data collection strategy with maximum delay constraints. On this basis, an adaptive recharging scheme based on maximum benefit is described in Section 5 and experimental results are shown in the next section.

## 2. Related Works

As mentioned before, in the era of big data and the Internet of Things, how to use one or more mobile elements for data collection as well as supplement the energy has become an important research topic. A large number of studies have shown that the routing strategy of MDCs and the recharging response mode of WCVs are the key factors to determine the network efficiency. In this section, the related works about these are described in detail.

### 2.1. Mobile Data Collection and Energy Replenishment in WRSN

Currently, there are three kinds of mobile Sink based data collection strategies:

- Random Mobility: The movement of MDC is on a random trajectory.
- Fixed Mobility: The MDC moves along a fixed pre-specified path.
- Controlled Mobility: The trajectory of MDC is controlled depending on its position and the density of data in its vicinity.

**Figure 1:**
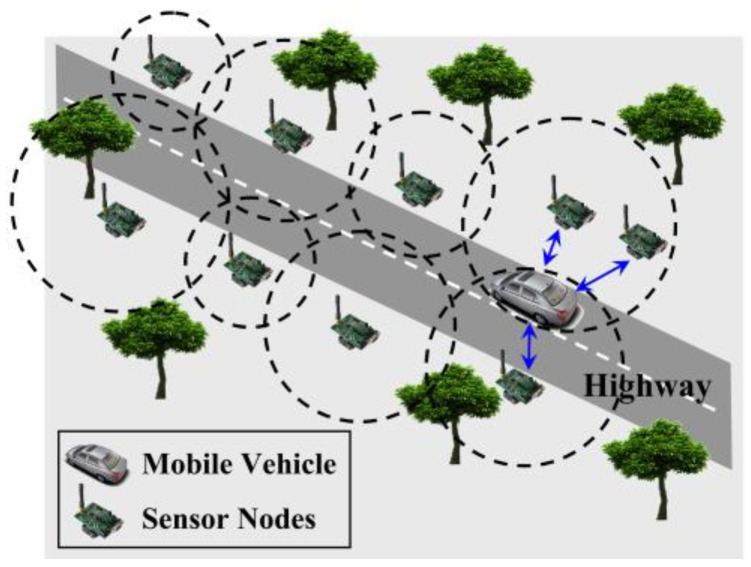
The mobile sink traverses the path to collect data from the one-hop sensor nodes [20].

**Figure 2:**
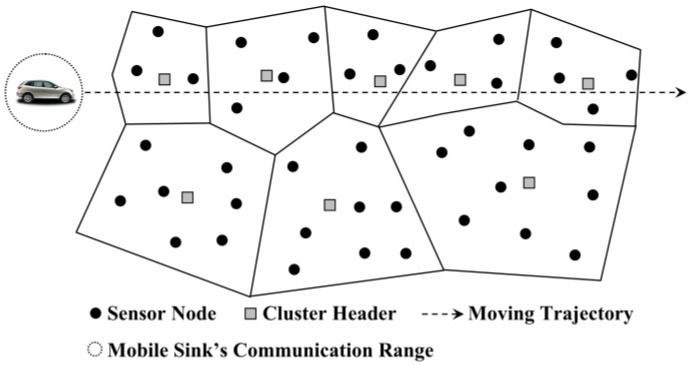
Unequal cluster formation in MobiCluster [21].

As an application example illustrated in Figure 1, sensor nodes are deployed along a highway to sense some statistical data related to traffic jams and then the line is patrolled by a mobile vehicle to collect this data efficiently [20]. Charalampos et al. [21] proposed another rendezvous-based sensory data collection approach, named MobiCluster. The mobile sink (MS) is mounted on public buses circulating within urban environments on fixed trajectories and near-periodic schedule. Nodes are often deployed in urban areas in proximity to public transportation vehicle routes. To balance energy consumption, nodes located near the sink trajectory are grouped in small-sized clusters while nodes located farther away are grouped into clusters of larger size (Figure 2). Compared to random mobility and fixed mobility, handling the mobility of sinks in a controlled manner is much more challenging [22]. In this mode, the MDC needs to continuously analyze and optimize its movement path according to the actual situation of the network. Although the random manner is relatively simple to implement, it has great uncertainty and high complexity of path optimization. At present, the data collection in WSNs is mostly periodic, so it is more appropriate to adopt the fixed mobility mode. However, fixed mobility is not flexible enough because of the real-time variation of node’s residual energy and the reconstruction of network topology. For this reason, the mobile data collection method with the help of the fixed traversal points and the controllable path is adopted in this paper.

**Figure 3:**
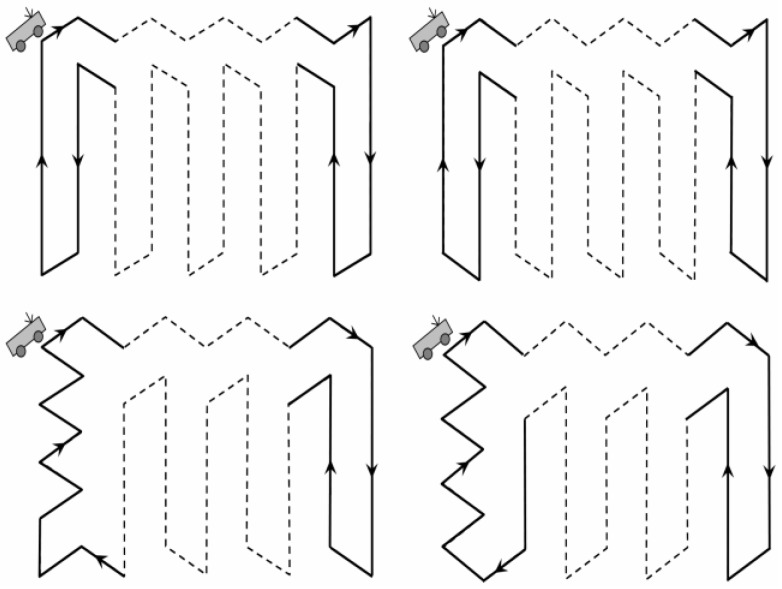
Different moving paths in [23].

**Figure 4:**
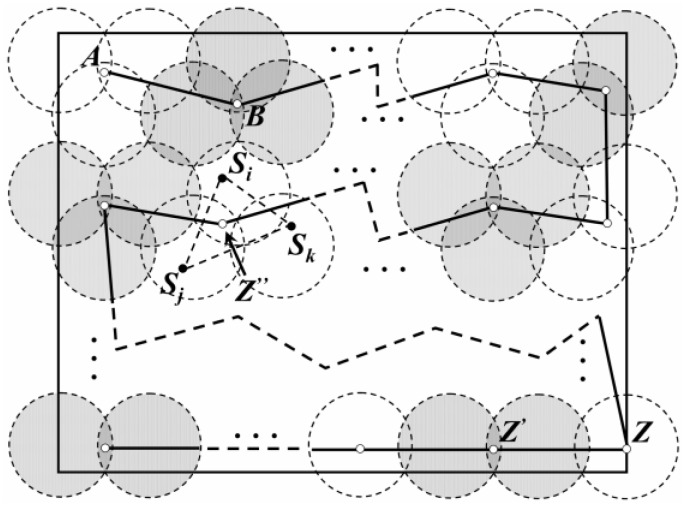
Trajectory of the mobile sink based on virtual regions [24].

A type of Data Gathering method based on one mobile sink moving along the Fixed traverse Points (DGFP) has been proposed in our previous work [23]. With the help of the sensing and coverage models, an optimal trajectory for the MS was built to achieve the balance of energy consumption (Figure 3). In addition, a sleep scheduling strategy has also been introduced to further reduce energy consumption. In [24], we have divided the network into several virtual regions and the leader of each region was selected according to its residual energy as well as the distance to all of the other nodes. A MS moves along the optimized path with a constant rate and communicated with each leader periodically, as shown in Figure 4. Then, it sends the collected data to the base station at the end of each data gathering cycle. However, the real-time of data collection in this method needs to be improved. For this reason, a type of Low-latency Data Gathering method with Multi-Sink (LDGM) has then been proposed in [25]. The leaders in each region communicated with several MSs which effectively reduced energy consumption and the end-to-end delay. Moreover, with the help of the sensing radius adjustment strategy, redundancy on network coverage was also effectively reduced. In addition, to reduce energy consumption on data collection, Gao et al. [26] have divided the sensor nodes into sub-sink nodes which were in a direct communication area or far-away nodes that were within the distance of the multi-hop communication area. Sinks move along the fixed path to gather data as much as possible.

It is worth noting that most MS-based data collection methods adopt a multi-hop way to upload data [3,4,7]. When the MS reaches a Cluster Header (CH) or rendezvous point, those data is upload centrally, otherwise, it is temporarily cached in these CHs or rendezvous points. Although this type of method improves the data collection efficiency in a distributed way, it is clear that the CH has a heavier load and higher energy consumption. In addition, the buffer overflow problem cannot be ignored. For example, to meet the requirements of the packet transmission delay and minimize the energy consumption of the whole network, a trajectory selection method based on priority of virtual points has been proposed in [27]. However, this method does not consider the buffer capacity of sensor node, so a high probability of buffer overflow may be occurred in practical application. Therefore, Gu et al. have improved it by dividing the network into multiple groups based on nodes’ locations and data generation rates [28]. In each group, the MDC visited each node at a fixed frequency to avoid buffer overflow.

With the deepening of research, replenishing energy while collecting data has gradually become a research hotspot. Zhao et al. [29] have proposed a standardized model for data collection and recharging nodes at the same time, but it was only applicable to smaller networks. Therefore, Dasgupta et al. [30] divided the network into multiple regions and used a MDC in each region to achieve data collection and wireless recharging. However, the wireless recharging rate is generally low nowadays due to the limitations of technology. For this reason, it is unreasonable to centralize data collection and recharging on one mobile device, which would result in a higher data gathering delay, data packet loss as well as poor network performance, especially in densely deployed network. Thus, in 2016 and 2017, Wang et al. [18] and Zhong et al. [31] have respectively proposed two types of data collection models by employing multiple MDCs and WCVs. Furthermore, Mehrabi et al. [20] and Khan et al. [32] have also put forward similar methods to avoid high delay during data collection caused by low recharging efficiency.

On the other hand, WCVs’ energy consumption while moving and their limited battery capacity are often ignored. For example, in the periodic mobile recharging scheme designed by Shi et al. [33], a method that reduced the recharging frequency of WCV by prolonging the sojourn time at the base station was proposed. However, it doesn’t make sense to assume that the energy carried by WCVs is infinite. Similarly, in [34], a starvation avoidance mobile energy replenishment scheduling method has been designed, which has effectively solved the energy starvation problem. However, the problem of energy finiteness of WCV is also not taken into account. In addition, at what time the node should send a recharging request to the WCV is also worth exploring. Many scholars believe that all nodes should follow a universally identical recharging threshold, in other words, the residual energy is the same when each node sends a request for recharging. However, the energy consumption rate is different due to the unequal loads and distances between nodes. Therefore, it is not appropriate to set a uniform recharging request threshold for all nodes. Also, for each node, the threshold can neither be too high nor too low. If the threshold is too high, the recharging request may be sent frequently, which may increase the time spend on moving and reduce the recharging efficiency of WCVs. On the contrary, if the threshold is too low, some nodes may fail to be recharged in time. Thus, they are more likely to die early, that affects the performance of the overall network. 

### 2.2. Path Planning about the Wireless Charging Vehicle

In WRSNs, how to plan the WCVs’ moving path reasonably and efficiently is important to the real-time and efficiency of recharging. Currently, there are three types of mobile path planning methods for WCV.

● Path Planning Based on Inquiring

At first, each node sends its own location information to the WCV, and then the WCV calculates a shortest path through all the nodes. Next, the WCV moves along the shortest path and checks the residual energy of each node. If it is lower than the preset recharging threshold, the node is recharged. A periodic recharging method based on this model was proposed in [35]. It has studied an optimization problem with the objective of maximizing the ratio of the WCV’s vacation time over the whole period of the recharging time. Other typical path planning methods based on inquiring include DPG-Scheme [36], RLT [37], etc. However, during its movement, if the residual energy of most nodes is higher, the WCV can only recharge a few nodes in one period, resulting in low recharging efficiency. In addition, there is only one WCV traversing all the nodes, so high recharging delay is unavoidable.

● Path Planning Based on Collaboration

The main idea is described as follows: low-energy nodes are selected as anchor nodes in the network, and other nodes transmit data to these anchor nodes in a one-hop or multi-hop manner. Subsequently, the WCV traverses these anchor nodes in turn and at the same time, recharges them as well as collects data from them. Other typical path planning strategies based on collaboration include ERDC [38], WerMDG [39], etc. However, in this type of schemes, the node with lower residual energy is always selected as the anchor node and its actual location does not been considered in path planning. This may cause uneven distribution of these anchor nodes. In addition, as mentioned above, integrating recharging and data collecting into one mobile device will inevitably affect the real-time performance of the network.

● On-Demand Path Planning

When a node’s residual energy is lower than the recharging threshold, a recharging request is sent to the WCV. This WCV then selects the most suitable node to recharge in sequence according to certain scheduling rules. Based on this method, a type of Nearest-Job-Next with Preemption strategy (NJNP) was proposed by He et al. in 2013 [40]. It achieves a high recharging efficiency by always selecting the nearest request node as the next recharging node. However, if the node that first requests for recharging is far away from the MCV, it is likely that this node will not be recharged during a long time due to preemption. In order to solve this unfair recharging problem, Feng et al. [34] have proposed a Starvation Avoidance Mobile Energy Replenishment scheme (SAMER) by calculating the maximum tolerable latency of each recharging requirement. To a certain extent, it avoids the premature death of nodes due to the failure of timely recharging. However, the battery capacity of WCV is considered to be infinite, which is inconsistent with the actual application. Zhu et al. [41] have taken the limited power of WCV into consideration and have proposed an Energy Starvation Avoidance Online Charging scheme (ESAOC). By calculating the maximum tolerable recharging delay and the shortest waiting time of nodes for recharging, the nodes which make the least number of other recharging-request nodes suffer from energy starvation are selected as the recharging candidates. Unfortunately, it does not further give a clear selection criterion when the number of the recharging candidate nodes is the same. In addition, most relevant researches require the WCV to judge whether it can return to the base station (BS) after each recharging. This is unwise because at the beginning of the recharging process, the residual energy of WCV is enough, and it needs not to make such a judgment every time. On the basis of analyzing all the above algorithms, a type of high-efficient Data Collection method based on Maximum Recharging Benefit in Sensor Networks (DCMRB) is proposed in this paper. 

## 3. Network Model

In this section, we construct the network architecture for distributed data collection and wireless recharging with the help of multi-MDC and multi-MCV. On this basis, we also describe the method for calculating the number of virtual traverse points.

### 3.1. Network Architecture

As mentioned before, if there is only one MDC and one WCV in the large-scale and densely deployed sensor network, there is a great possibility that data collection and energy supplement may be unable to be completed. On the one hand, both MDCs and WCVs move at a relatively slow speed, and they need to stay at each node for some time during the process of data collection and wireless recharging, which increases the time delay. Moreover, the wireless recharging rate of sensor node is still slow. Although recharging one node does not need much time, a mass of node recharging requests will inevitably affect the real time of the network. This may cause nodes death because some of them may not be recharged in time. Hence, it is also unrealistic to integrate MDC and WCV together. In view of this, we divide the network into several regions, and a MDC as well as a WCV moves in each region for data collection and wireless recharging. Moreover, in order to further enhance the efficiency of data collection, each MDC only needs to stay at the Virtual Traverse Point (VTP) without having to traverse each node. 

Without loss of generality, it is assumed that *N* rechargeable nodes are uniformly and randomly deployed in a rectangular region whose length and width are *M* and *L*, respectively. *R_S_* and *R_t_* are defined as the sensing and communication radius of each node, respectively, and a stationary BS is located at the center of the network. To ensure completely coverage in an omni-directional sensor network, the maximum and minimum density of nodes can be expressed as follows [23]:

(1) $$\rho_{\max}=2/\sqrt{3}R_{s}^{2}$$

(2) $$\rho_{\min}=2/3\sqrt{3}R_{s}^{2}$$

**Figure 5:**
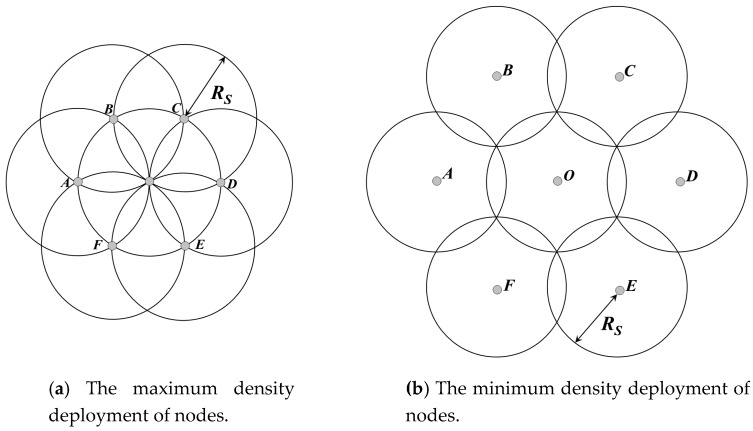
The maximum and minimum density deployment of nodes.

As shown in Figure 5a,b, when the density of nodes is higher than *ρ*_max_, redundant nodes exist in the network [23]. On the contrary, if it is lower than *ρ*_min_, the coverage hole appears. In general, the density of nodes (*ñ*) in this paper is set to *(ρ*_max_ + *ρ*_min_)/2.

**Figure 6:**
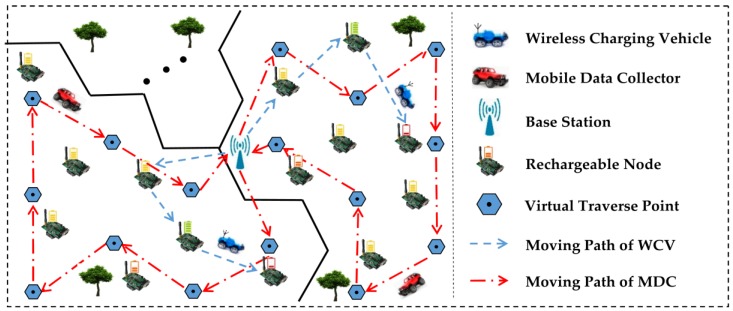
Network architecture.

The network is divided into *k* regions that are approximately equal in size. The principle of this division is described in Section 4.2 and we call it “Network Partition Method based on Virtual Scan Line” (VSL). In each region, a MDC (the red car in Figure 6) and a WCV (the blue car equipped with wireless recharging device in Figure 6) moves in this field to collect data or recharge nodes. In addition to collecting, processing and storing the sensing data of the entire network, the base station is also responsible for replacing the battery of the MDC and the WCV. The overall network architecture is shown in Figure 6.

Let’s take a region as an example. At the beginning of each round of data collection, the MDC in this region departs from BS. When it arrives at a VTP, it receives data uploaded from nodes around it. The MDC won’t move to the next VTP until all data in those nodes has been completely collected. To avoid increasing time delay on data collection, the nearest neighbor discovery algorithm [22] is adopted to build the shortest moving path of MDC in each region, as indicated by the red dotted arrow in Figure 6. After traversing all the VTP in the region, the MDC finally returns to BS and uploads the data it collects. On the other hand, each node calculates a “Threshold of Recharging Request” (TRR) based on its own energy consumption rate. When the residual energy of one node is below the TRR, the recharging request is sent to the WCV immediately. Then, according to the proposed wireless recharging scheme, the WCV selects the most suitable recharging node to supplement its energy, so as to avoid the death of the node and prolong the network lifetime. The moving path of the WCV is shown by the blue dotted arrow in Figure 6. 

Based on this architecture, the key problems that need to be solved in this paper are described as follows:

(1) How to get the minimum number of MDCs and how to design a reasonable and feasible data collection scheme under the constraint of network size, number of nodes as well as time delay to achieve real-time and efficient data collection with balanced energy consumption;
(2) How to properly divide the network into regions based on the minimum number of MDCs;
(3) How to set a reasonable recharging threshold for each node and formulate an optimal recharging scheme under the condition of limited battery capacity of WCV, so as to recharge the requested nodes in time and maximize the recharging efficiency.

Therefore, a type of high-efficient Data Collection method based on Maximum Recharging Benefit (DCMRB) is proposed in this paper. Firstly, the complete coverage model in WSNs is adopted to determine the number and specific distribution of VTPs. Secondly, the minimum number of MDCs is calculated out under the constraints of time delay and buffer overflow. Based on these, the Virtual Scan Line (VSL) method is proposed to evenly divide the network, and then, the moving speed of MDCs is optimized in order to ensure the integrity of data collection. Moreover, in view of the fact that the existing WCV recharging scheme cannot fully meet the recharging requirements of nodes with different energy consumption rates, an adaptive calculation method of the recharging request threshold is proposed. The definitions of parameters about the network are shown in Table 1. 

### 3.2. Virtual Traverse Points

For WSNs that employing MDCs for data collection, the moving path of the MDC should be firstly considered. Similar to [23], some uniformly distributed Virtual Traverse Points (VTPs) are set in the network. During each round of data collection, the MDC receives data uploaded from the nearby nodes only when it arrives at each VTP. Additionally, the following constraints need to be met:

(1) When the MDC arrives at the VTP, it can only receive data uploaded by nodes located in its communication region.
(2) Each sensor node in the network should have the opportunity to upload data to the MDC within one hop.
(3) In order to avoid repeated collection of redundant data and to enhance the real-time performance, MDCs are only allowed to pass through each VTP once and only once during a round of data gathering time.

**Figure 7:**
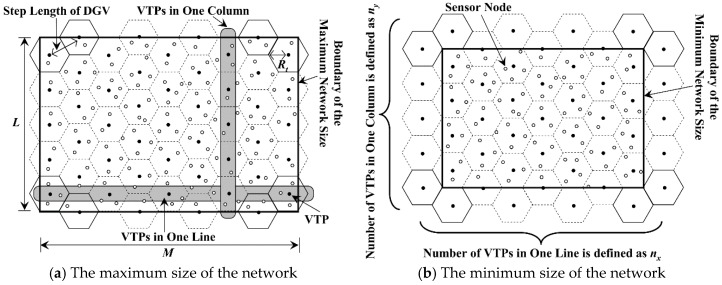
Distribution of the virtual traverse points.

Thus, the location of each VTP should firstly be calculated out. As shown in Figure 7, the network is divided into a number of regular hexagons (RHs), whose side length is *R_t_*. The center of each RH (black dots in Figure 7) is defined as the Virtual Traverse Point (VTP). When the number of RHs is fixed, the maximum and the minimum sizes of the network are shown in Figure 7a,b, respectively.

Thus, according to the first and the second constraints mentioned above, the step length of MDC is set to $\sqrt{3}R_{t}$. For the convenience of discussion, the total number of VTPs in the network is set to *m*. Moreover, *n*_x_ and *n*_y_ are defined as the number of VTPs in one line and one column, respectively. It is known from Figure 7 that, the value of *n*_x_ can be calculated by Equation (3). Meanwhile, we define a temporary variable *q*_y_ in Equation (4), which is an important parameter for calculating the value of *m*:

(3) $$n_{x}=\left\lceil \left(M/0.5R_{t}+1\right)/3\right\rceil$$

(4) $$q_{y}=\left\lceil 2L/\sqrt{3}R_{t}\right\rceil$$

To ensure that the data can be uploaded to MDC within one hop, the network should be completely covered by RHs. However, for the networks with the same number of VTPs, their sizes may be different, as shown in Figure 7a,b. Based on the analysis above as well as the relationship between *M*, *L* and *R_t_*, the total number of VTPs can be calculated out in the following three cases.

**Figure 8:**
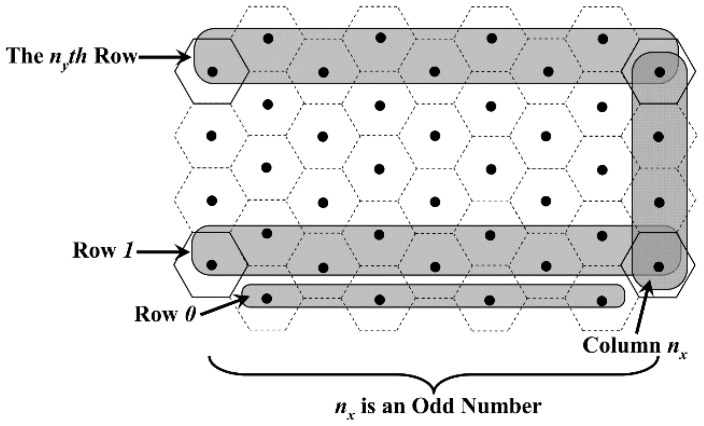
Distribution of VTPs when *q*_y_ is odd.

Case 1: When *q*_y_ is odd, according to Figure 8, whether *n*_x_ is odd or even, we have:

(5) $$n_{y}=\left(q_{y}+1\right)/2$$

that is:

(6) $$m=n_{x}\times \left(\frac{q_{y}+1}{2}\right)=\left\lceil \left(\frac{M}{0.5R_{t}}+1\right)/3\right\rceil \times \frac{1}{2}\left(\left\lceil \frac{2L}{\sqrt{3}R_{t}}\right\rceil +1\right)$$

**Figure 9:**
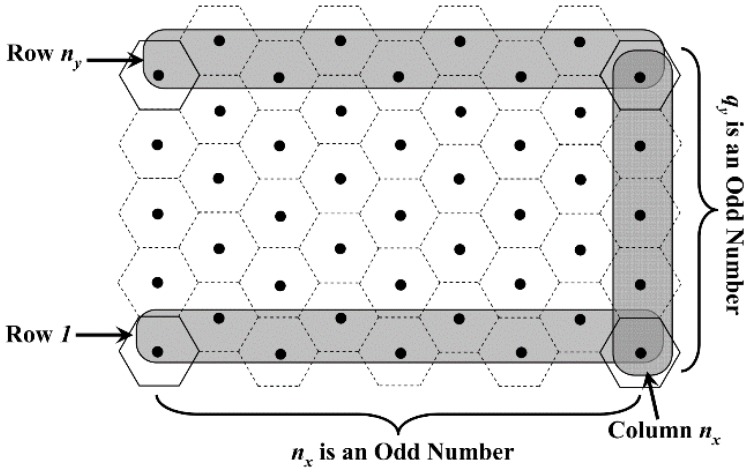
Distribution of VTPs when both *q*_y_ and *n*_x_ are even numbers.

Case 2: When *q*_y_ and *n*_x_ are all even, according to the above definitions and Figure 9, the number of VTPs in each column can be described as follows:

(7) $$n_{y}=\{\begin{array}{c} q_{y}/2\;\mathrm{for}\;\mathrm{the}\;\mathrm{odd}\;\mathrm{column}\\ q_{y}/2+1\;\mathrm{for}\;\mathrm{the}\;\mathrm{even}\;\mathrm{column} \end{array}$$

so:

(8) $$m=\frac{n_{x}}{2}\times \frac{q_{y}}{2}+\frac{n_{x}}{2}\times \left(\frac{q_{y}}{2}+1\right)=n_{x}\times \left(\frac{q_{y}+1}{2}\right)=\left\lceil \left(\frac{M}{0.5R_{t}}+1\right)/3\right\rceil \times \frac{1}{2}\left(\left\lceil \frac{2L}{\sqrt{3}R_{t}}\right\rceil +1\right)$$

The value of *m* is equal to that in case 1.

**Figure 10:**
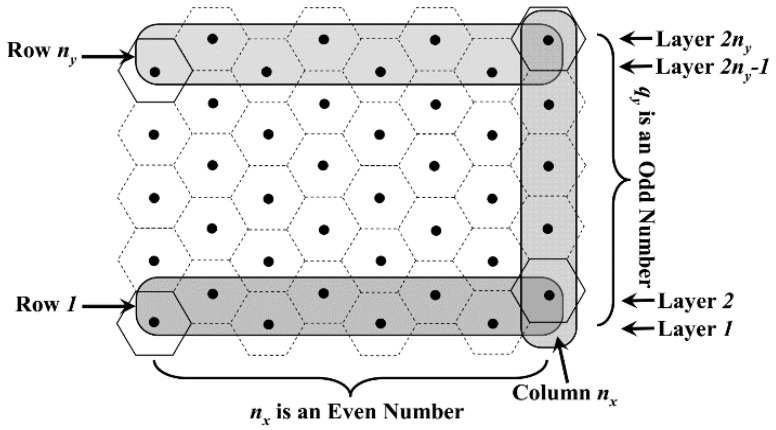
Distribution of VTPs when *q*_y_ is even and *n*_x_ is odd.

Case 3: When *q*_y_ is an even number and *n*_x_ is an odd number, according to Figure 10, the value of *n*_y_ is still in accordance with Equation (7). However, in this case, the number of VTPs in each odd column is one more than that in each even column. Thus, it is obvious that the value of *m* is:

(9) $$m=\left(\frac{n_{x}+1}{2}\right)\times \frac{q_{y}}{2}+\left(\frac{n_{x}-1}{2}\right)\times \left(\frac{q_{y}}{2}+1\right)=n_{x}\times \left(\frac{q_{y}+1}{2}\right)-\frac{1}{2}=\left\lceil \left(\frac{M}{0.5R_{t}}+1\right)/3\right\rceil \times \frac{1}{2}\left(\left\lceil \frac{2L}{\sqrt{3}R_{t}}\right\rceil +1\right)-\frac{1}{2}$$

## 4. Multi-MDCs Based Data Collection Strategy with Maximum Delay Constraint

In this section, we describe how to calculate the number of required MDCs under the constraint of maximum time delay on data transmission. On this basis, a network partitioning method based on virtual scan line is then proposed. In addition, in order to avoid buffer overflow during data collection, we also propose a speed adjustment scheme for MDC. The definitions of parameters in this section are shown in Table 2. 

### 4.1. The Minimum Number of MDCs that Meets the Delay Constraint

According to the network model and the characteristic about mobile data gathering, the following constraints need to be met in DCMRB:

(1) Data collected from any node in the network must be transmitted to BS within the time period, *T_d_*. In other words, the constraint of the maximum time delay needs to be met.
(2) Each VTP needs to be accessed by one MDC only once during the time period, *T_r_*.
(3) During each round of data collection, the MDC needs to depart from BS and eventually return to BS.
(4) The MDC can only stay at the BS or the VTPs. Moreover, the regular hexagons where the MDC stays at for two consecutive times must be adjacent.
(5) The MDC moves linearly between two consecutive VTPs, and its speed is *v*_MDC_.

It is known that, the density of nodes in the network is *N*/(*M* × *L*). Therefore, the number of nodes in any *RH_i_* (*i* = 1, 2, …, *m*) can be approximately calculated as follows:

(10) $$Num\left(RH_{i}\right)=\left(3\sqrt{3}R_{t}^{2}\times N\right)/\left(2M\times L\right)$$

Without loss of generality, the time duration that the MDC stays at each VTP is defined as *t_s_*. In addition, the sensing rate and the data uploading rate of each node are set to *g bit*/*s* and *u bit/s*. Hence, in order to ensure the integrity of data collection, the minimum value of *t_s_* should be:

(11) $$t_{s}=Num\left(RH_{i}\right)\times g\times T_{r}/u$$

According to Equations (10) and (11) can be rewritten as follows:

(12) $$t_{s}=\left(3\sqrt{3}R_{t}^{2}\times N\times g\times T_{r}\right)/\left(2u\times M\times L\right)$$

**Figure 11:**
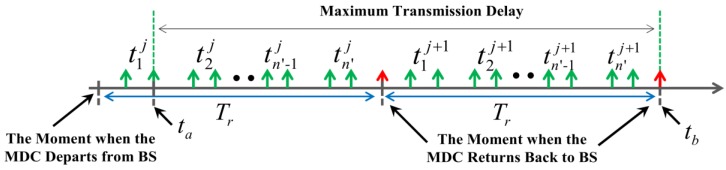
Maximum delay on data packet transmission.

Furthermore, we discuss the maximum possible transmission delay in this data collection mode. As shown in Figure 11, $t_{i}^{j}$ is defined as the time duration when the MDC stays at the *i*th VTP in the *j*th round of data collection. *t_a_* is regarded as the moment when the MDC just left the first VTP. Moreover, *t_b_* is defined as the moment when the MDC arrives at the base station at the end of the (*j* + 1)th round of data collection, so if a node in the first RH generates a data packet at *t_a_* (at this moment, the MDC has just left this RH), this packet can only be delivered to BS at *t_b_*. 

According to the analysis above, the maximum delay on data packet transmission (defined as $T_{d}^{\max}$) can be expressed by Equation (13). $d_{BS\;to\;RH_{i}}/v_{\mathrm{MDC}}$ is the time spending on moving from BS to the first VTP, and *t_s_* is the time for the MDC to stay at this VTP:

(13) $$T_{d}^{\max}=2T_{r}-d_{BS\;to\;RH_{i}}/v_{\mathrm{MDC}}-t_{s}$$

In Equation (13), $d_{BS\;to\;RH_{i}}$ is the Euclidean distance between BS and the first VTP. Normally, it is assumed that the value of it is $\sqrt{3}R_{t}$. To ensure the integrity of data collection, $T_{d}^{\max}$) should not be greater than *T_d_*. That is:

(14) $$T_{d}^{\max}=2T_{r}-\sqrt{3}R_{t}/v_{\mathrm{MDC}}-t_{s}\leq T_{d}$$

Combining Equations (12) and (14), the first condition that *T_r_* needs to meet can be obtained as follows:

(15) $$T_{r}\leq \left(T_{d}+\frac{\sqrt{3}R_{t}}{v_{\mathrm{MDC}}}\right)/\left(2-\frac{3\sqrt{3}R_{t}{}^{2}\times N\times g}{2u\times M\times L}\right)$$

Additionally, it is also necessary to ensure that the amount of data collected by a sensor node during *T_r_* does not exceed its buffer size (defined as *C*). Thus, the second constraint that *T_r_* needs to meet is:

(16) $$T_{r}\leq C/g$$

To meet the constraint of time delay as well as to avoid buffer overflow, the maximum value of *T_r_* should be the smaller value of the two upper limits in Equations (15) and (16). That is:

(17) $$Min\left(\left(T_{d}+\frac{\sqrt{3}R_{t}}{v_{\mathrm{MDC}}}\right)/\left(2-\frac{3\sqrt{3}R_{t}{}^{2}Ng}{2uML}\right),\;C/g\right)$$

In this case, assuming a MDC traverses up to *m*′ VTPs during *T_r_*. The time spending on data collection and moving are $\left(m^{\prime }-1\right)t_{s}$ and $\left(\left(m^{\prime }-1\right)\sqrt{3}R_{t}+d_{RH_{m}\;to\;BS}\right)/v_{\mathrm{MDC}}$, respectively. So:

(18) $$T_{r}+\left(m^{\prime }-1\right)t_{s}+\left(\left(m^{\prime }-1\right)\sqrt{3}R_{t}+d_{RH_{m}\;to\;BS}\right)/v_{\mathrm{MDC}}\leq T_{d}^{}$$

Here, $d_{RH_{m}\;to\;BS}$ is defined as the Euclidean distance between the *m*′th VTP and the base station. In the worst case, we have:

(19) $$d_{RH_{m^{\prime }}\;to\;BS}=\sqrt{{\left(M/2\right)}^{2}+{\left(L/2\right)}^{2}}$$

By combining Equations (14), (18) and (19), it is known that:

(20) $$m^{\prime }\leq \frac{v_{\mathrm{MDC}}\left(T_{r}+t_{s}\right)-t_{s}-\sqrt{{\left(M/2\right)}^{2}+{\left(L/2\right)}^{2}}}{v_{\mathrm{MDC}}t_{s}+\sqrt{3}R_{t}}$$

So, the minimum number of MDCs required in the network is:

(21) $$k=\left\lceil \frac{m}{m^{\prime }}\right\rceil =\left\lceil \frac{m\left(v_{\mathrm{MDC}}t_{s}+\sqrt{3}R_{t}\right)}{v_{\mathrm{MDC}}\left(T_{r}+t_{s}\right)-t_{s}-\sqrt{{\left(M/2\right)}^{2}+{\left(L/2\right)}^{2}}}\right\rceil$$

### 4.2. Network Partition Method Based on Virtual Scan Line

**Figure 12:**
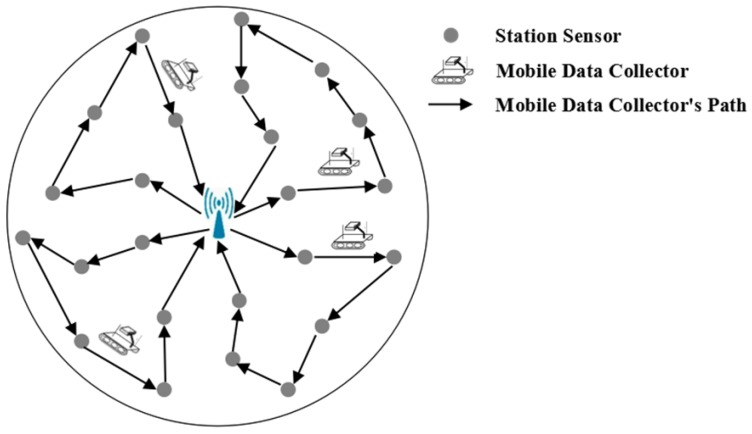
Network partition strategy of DCEM [30].

According to the minimum number of MDCs under the constraints of maximum transmission delay and no packet loss, the network can be further divided into *k* regions. In each region, there is only one MDC for data collection. Some related studies have been carried out. Dasgupta et al. [30] have proposed a type of Delay-Constrained Energy Minimization based data gathering algorithm (DCEM). A circular network was divided into four regions, and in each region, the moving paths of the mobile data collectors were built in a balanced way, as shown in Figure 12. However, DCEM does not consider the possibility of buffer overflow, and it is only applicable to the network where nodes are uniformly deployed. According to the node’s dynamic variability of sensing rate, Wang et al. [18] have regarded the data generation process as a Poisson process to estimate the required number of MDCs. Then, the network has been divided into several regions with the help of the k-means method that greatly balances energy consumption and reduces data collection latency. Nevertheless, when the sensing mode changes, this method is not fully applicable. In [31], the rectangular network has been divided into several parts according to the number of MDCs. Subsequently, a twice-partition algorithm based on center points and an Anchor Selection algorithm based on the tradeoff between Neighbor Amount and residual Energy (AS-NAE) have been proposed respectively to handle the complex scheduling problem of multiple vehicles. As a result, the data transmission delay and energy consumption of MDCs were reduced. Although this network partition strategy is more reasonable, the time complexity of it is still higher.

So, how to divide the network into regions to balance the workload of each MDC as much as possible is an important factor that determines the effect of distributed mobile collection. As mentioned before, in DCMRB, VTPs are uniformly distributed in the network, and the MDC traverses each of them in turn. Therefore, a partition method based on Virtual Scan Line (VSL) is proposed.

Firstly, we discuss the most suitable number of VTPs in each partition. As mentioned earlier, assuming that the total number of VTPs in the network is *m* and the network is divided into *k* areas. So, the following two cases are considered:

Case 1: If *mod (m*/*k)* == 0, it is considered that the distributed data collection will show better balance when the number of VTPs in each partition is *m*/*k*.

Case 2: If *mod (m*/*k)* ≠ 0, it is considered that the balance of DCMRB will be better when each of the *mod (m*/*k)* partitions contain *m*/*k* + 1 VTPs, and each other partition contains *m*/*k* VTPs. 

**Figure 13:**
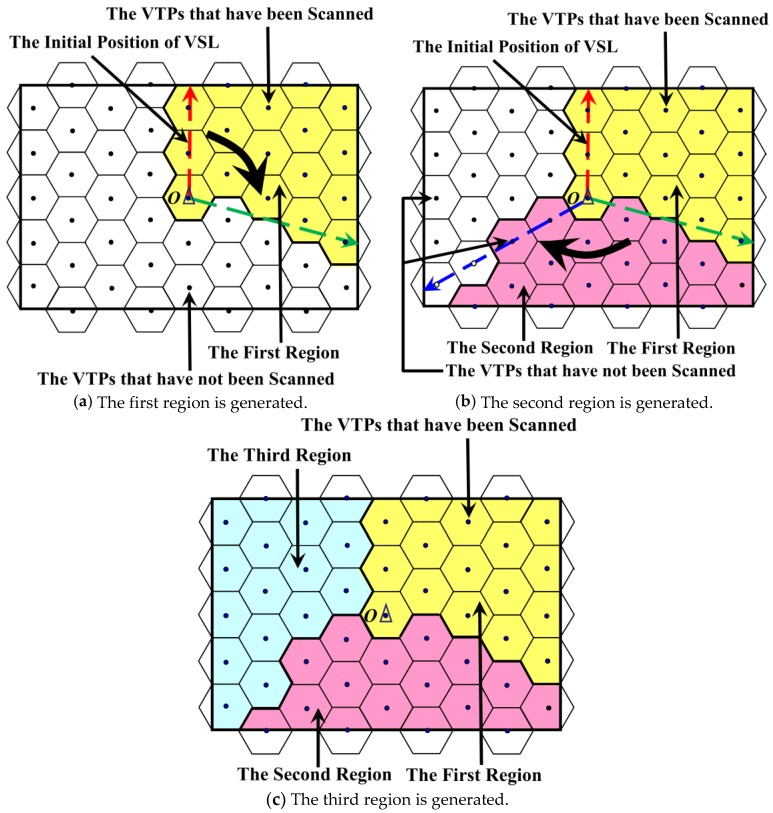
The network partition based on virtual scan line.

The process of the partition method based on Virtual Scan Line is shown in Figure 13. It is worth mentioning that this method is not affected by the network size and the values of *n*_x_ and *q*_y_. There are 49 VTPs in the network, and we assume that the number of MDCs is three (calculated by Equation (21)). That is, the network needs to be divided into three regions. Due to the fact that *mod (49/3) = 1* and *49/3 = 16*, the number of VTPs in these three regions should be 17, 16 and 16, respectively. The base station is located at the center of the network (denoted as *O*) and overlaps with the location of one VTP. It is worth mentioning that, whether or not the location of BS overlaps with that of VTP, the effect of DCMRB is not affected.

Initially, *O* is the starting point of the red dotted line, and this line (named as “Virtual Scan Line”, VSL) intersects the boundary of the rectangular network vertically. VSL is centered at point *O* and rotates clockwise to scan the VTPs in the network. All VTPs are initially “unscanned”. When a VTP is scanned by this VSL, its status is changed to “scanned”. When the number of VTPs newly scanned by this VSL is greater than or equal to the expected number of VTPs in the corresponding region, the scanning process is paused and a region is generated according to the following two cases. Then, the VSL starts from the paused position of the last scan and carries out the next scan until the same pause condition is encountered. When all the regions of the network are formed, the scanning process is completely over. 

There are two cases in the process of network partition:

Case 1: During one scanning process, if the number of VTPs scanned by the VSL is exactly equal to expected in the corresponding partition, those RHs that the scanned VTPs located in are divided into the same region. For example, in Figure 13a, when the 17th VTP is scanned by the green dotted line, the number of the scanned VTPs during this scan is equal to the expected value. Therefore, the RHs that those 17 VTPs located in form the first region of the network, as the yellow area shows in Figure 13a.

Case 2: If the number of VTPs scanned is greater than expected in the corresponding region, the scan is paused immediately. At the same time, based on the distance from the base station, the VTPs on the VSL now are marked as “unscanned” successively from far to near, until the number of VTPs marked as “scanned” meets the expected requirement. For example, in Figure 13b, the VSL scans from the green dotted line to the blue dotted line, and 18 new VTPs are scanned during this period. What needs to be pointed out is that the VTPs at the green dotted line have been scanned in the previous scanning process, so they are not regarded as the new VTPs this time. However, the most appropriate number of VTPs in this region is 16. Therefore, at the current location of VSL, the two VTPs farthest from the base station (the white dots) are remarked as “unscanned”. Thus, the second region is formed, as the pink area shows in Figure 13b.

Finally, the network is divided into *k* regions after finishing the scanning process (Figure 13c), and the number of VTPs in each region is almost the same. 

### 4.3. Region Size Adjustment

**Figure 14:**
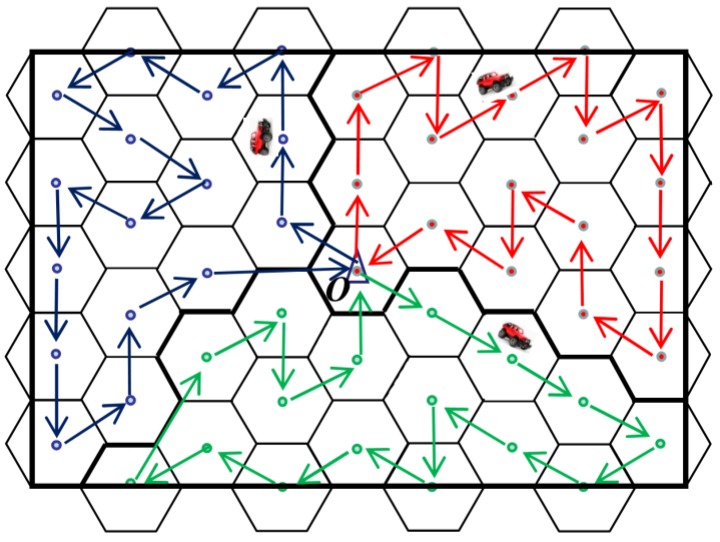
The shortest moving path of MDC in each region.

Although the network is approximately evenly divided into several regions with the help of the VSL, it is also necessary to ensure that *T_r_* is not greater than the value described in Equation (17). To solve this problem, the nearest neighbor discovery algorithm [22] is first adopted to build the shortest path of MDC in each region, as shown in Figure 14.

*D_i_* and $Num_{i}^{\mathrm{VTP}}$ represent the path length of MDC and the number of VTPs in the *i*th region (*i* = 1, 2, …, *k*), respectively. Therefore, the data collection cycle (denoted as *T_i_*) of this region after adjustment can be expressed as follows:

(22) $$T_{i}=D_{i}/v_{\mathrm{MDC}}+Num_{i}^{\mathrm{VTP}}\times t_{s}$$

Then, by considering the relationship between the maximum value (i.e., *T_j_*) that taken from *T*_1_, *T*_2_, …, *T_k_* and Equation (17), we get:

(1) If $T_{j}\leq Min\left(\left(T_{d}+\frac{\sqrt{3}R_{t}}{v_{\mathrm{MDC}}}\right)/\left(2-\frac{3\sqrt{3}R_{t}{}^{2}Ng}{2uML}\right),\;C/g\right)$, the current network partitions need not to be changed. Thus, *k* is the final number of the regions as well as the number of the MDCs.

(2) If $T_{j}>Min\left(\left(T_{d}+\frac{\sqrt{3}R_{t}}{v_{\mathrm{MDC}}}\right)/\left(2-\frac{3\sqrt{3}R_{t}{}^{2}Ng}{2uML}\right),\;C/g\right)$, there is at least one region cannot meet the constraints described in Section 4.1. That is to say, the network partition is not reasonable. Hence, let *k* = *k* + 1 and carry out the VSL based partitioning method again until the first condition is met. 

### 4.4. Speed Adjustment Scheme for Ensuring Data Integrity

As mentioned before, we assume that the sojourn time for MDC at each VTP is *t_s_*. However, in DCMRB, the number of nodes in each RH is not exactly equal to each other because they are uniformly and randomly distributed, especially for those RHs that are located at the boundary of network. Therefore, if the value of *t_s_* is calculated by Equation (12), packet loss may be happened because sensing data generated in some RHs cannot be completely transmitted to MDC in *t_s_*. In this case, the moving speed of MDC should be increased.

For the *i*th region, let *D* (*j* − 1, *j*) denote the Euclidean distance between two adjacent VTPs (VTP*_j_*_−1_ and VTP_j_) in the MDC’s moving path. In particular, the base station is assumed to be VTP_0_. Thus:

(23) $$t\left(j-1,j\right)=D\left(j-1,j\right)/v_{\mathrm{MDC}}+t_{s}$$

**Figure 15:**
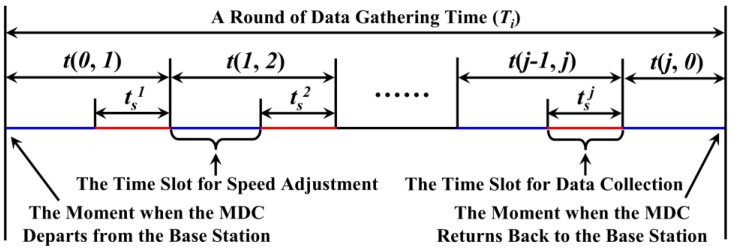
The sequence diagram about data collection and speed adjustment in *T_i_*.

Here, *t* (*j* − 1, *j*) is referred to “Unit Data Collection Period (UDCP)” of MDC, as shown in Figure 15. According to Equation (23), it is not difficult to know that:

(24) $$T_{i}=\sum_{j=1}^{Num_{i}^{\mathrm{VTP}}}t\left(j-1,j\right)+D\left(j,0\right)/v_{\mathrm{MDC}}$$

As mentioned above, it is not completely reasonable that the sojourn time for the MDC to stay at each VTP be the same. However, the time length of a round of data collection and the number of regions have been calculated out based on this assumption. In order to ensure the integrity of the collected data, the sojourn time and moving speed of MDC are further adjusted while keeping the value of UDCP unchanged.

Let $t_{s}^{j}$ denote the shortest time duration during which the MDC stays at VTP*_j_* without losing packets. It is not difficult to know that:

(25) $$t_{s}^{j}=Num\left(RH_{j}^{i}\right)\times g\times T_{i}/u$$

In the *i*th region, $Num(RH_{j}^{i})$ is regarded as the total number of nodes in the RH where VTP*_j_* is located at. If $t_{s}^{j}$ ≤ *t_s_*, let $t_{s}^{j}$ = *t_s_*, and we do not change the speed of MDC. If $t_{s}^{j}$ > *t_s_*, the moving speed of the MDC from VTP*_j_*_−1_ to VTP_j_ (defined as *v*_MDC_ (*j* − 1, *j*)) is modified by the following equation:

(26) $$v_{\mathrm{MDC}}\left(j-1,j\right)=D\left(j-1,j\right)/\left(t\left(j-1,j\right)-t_{s}^{j}\right)$$

That is to say, by accelerating its moving speed, the MDC has enough time to collect data. In summary, the sequence diagram of a MDC (located in the *i*th region) in one round of data collection time is shown in Figure 15. 

## 5. An Adaptive Recharging Scheme Based on Maximum Benefit

With the help of the MDCs in each region, the problems of uneven load and the large difference in energy consumption among nodes are greatly alleviated. However, continuous sensing and periodic data uploading still accelerate the energy consumption rate of nodes, especially for those nodes that need to collect multimedia data. Thus, to ensure that the network runs steadily for a long time, several Wireless Charging Vehicles (WCVs) are assigned into the network. After receiving the recharging requests, the WCV will select the most suitable nodes for recharging according to certain rules to avoid node death due to energy depletion. Definitions of the related parameters are listed in Table 3.

### 5.1. Energy Threshold of the Recharging Request

In WRSN, when the residual energy of one node is below the “Threshold of the Recharging Request (TRR)”, the recharging request is sent to the WCV immediately. In many studies, the value of TRR is equal for each node. However, this is a little unreasonable. On one hand, the distances between nodes and VTPs may be different from each other, so are the energy consumption on transmission. On the other hand, if the threshold is too high, the recharging request may be sent frequently, which will prolong the time on moving and reduce the recharging efficiency of WCV. On the contrary, if the threshold is too low, some nodes may fail to be recharged in time and die, thus affects the overall network performance. 

To solve this problem, Wang et al. [18] have proposed a type of solution method about the adaptive TRR. According to the distance between nodes and BS, the network was divided into several coronas. Then, the value of TRR was proportional to the average energy consumption rate of nodes in each corona. Although the difference on energy consumption between coronas is fully considered in this method, it is still a little unreasonable that the TRRs are set to the same value in the same corona. Even for nodes in the same corona, the load on them is also likely to be different from each other.

In DCMRB, although the nodes only need to send data to the MDC within one hop, the transmission distance may also be different due to their uniformly and randomly distribution, which causes the unbalanced energy consumption. Therefore, a calculation method about the adaptive value of TRR is proposed in this paper:

(1) Energy Consumption Rate

There are two types of attenuation models of wireless signal, that are free-space model and multi-path fading model. In Wireless Sensor Networks, the most commonly used method to calculate energy consumption on communication is Equation (27), which was proposed by Heinzeman [42]:

(27) $$E_{send}\left(q,d\right)=\{\begin{array}{c} qE_{elec}+q\varepsilon_{fs}d^{2}\;d<d_{0}\\ qE_{elec}+q\varepsilon_{fs}d^{4}\;d\geq d_{0} \end{array}$$

Here, *E_elec_* is the unit energy consumption of the sending and receiving circuit. *ε_fs_* is the energy consumption of amplifier in free-space model, and *q* is the amount of data that need to be sent. Let *d*_0_ denote the threshold of distance, which is generally equivalent to the value of *R_t_*. Therefore, the energy consumption rate of node *i* can be expressed as follows:

(28) $$P_{i}=e_{s}+g\times \left(E_{elect}+\varepsilon_{fs}\times d_{i\;\mathrm{to}\;\mathrm{VTP}}^{2}\right)$$

In Equation (28), *d_i_*_to VTP_ is the Euclidean distance between node *i* and the VTP nearest to it. In addition, *e_s_* is defined as the energy consumption rate on sensing.

(2) The Number of Nodes that a WCV can Recharge during a Round of Recharging Time

Without loss of generality, in DCMRB, the WCV needs to arrive to the node to recharge it “one-to-one”, until the node’s energy reaches to *C_b_* again. For simplicity, the effect of distance on recharging efficiency is ignored. Let *N_j_* denote the total number of nodes in the *j*th region and their IDs are noted as 1, 2, 3, …, *N_j_*, respectively. Besides, the ID of the base station is defined as 0. So, the average distance between any two nodes (including the base station) in this region (denoted as $\bar{d_{j}}$) is:

(29) $$\bar{d_{j}}=\frac{1}{N_{j}\times \left(N_{j}+1\right)}\sum_{x=0}^{N_{j}}\sum_{y=0,y\neq x}^{N_{j}}d_{xy}$$

Here, *d_xy_* is the distance between node *x* and node *y*. Therefore, the number of nodes that can be recharged in a round of recharging time is:

(30) $$N_{j}^{\prime }=\left\lfloor \left(C_{h}-\left(N_{j}^{\prime }+1\right)\times \bar{d_{j}}\times e_{m}\right)/C_{b}\right\rfloor$$

*e_m_* is defined as the energy consumption of WCV on travelling one meter. *C_b_* and *C_h_* are the battery capacities of a sensor node and the WCV, respectively.

(3) The Value of TRR

According to the analysis above, the time duration during which the WCV finishes a round of recharging is expressed as $\left(C_{b}/\eta \right)\times N_{j}^{\prime }+\left(N_{j}^{\prime }+1\right)\times \bar{d_{j}}/v_{\mathrm{WCV}}$. *η* and *v*_WCV_ are defined as the wireless recharging rate and the moving speed of WCV, respectively. To meet all the recharging requests in this region, the WCV needs to carry out at least $\left\lceil N_{j}/N_{j}^{\prime }\right\rceil$ rounds of recharging. Hence, in the worst case, the duration from when a node sends a recharging request to the time it is recharged is:

(31) $$T_{j}=\left\lceil N_{j}/N_{j}^{\prime }\right\rceil \times \left(C_{b}/\eta \times N_{j}^{\prime }+\left(N_{j}^{\prime }+1\right)\times \bar{d_{j}}/v_{\mathrm{WCV}}\right)$$

In summary, the threshold of recharging request for node *i* is *P_i_ × T_j_*. That is to say, if the residual energy of node *i* reaches or falls below this value, a recharging request should be sent to WCV immediately, otherwise it may die because of untimely recharging.

### 5.2. Wireless Recharging Scheme Based on Limited Battery Capacity and the Maximum Recharging Benefit

At the beginning, the WCV is located at BS. When it receives the recharging requests from nodes, their IDs are added to the “Recharging Request Queue” (denoted as *Ф*). For the WCV, the simplest recharging scheme is called “Greedy Mobile Scheme based on Maximum Recharging Benefit (GMS-MRB)”. To be specific, if the residual energy of WCV is sufficient for it to return to BS, the node with the highest recharging benefit will always be recharged. The recharging benefit of node *i* is defined as *R_i_*/*E*_WCV to_ *_i_*, where *R_i_* indicates the amount of energy that node *i* needs to be recharged. Obviously, the maximum value of *R_i_* is *C_b_*. Besides, the energy consumption of WCV on moving from the current position to node *i* is *E*_WCV to *i*_.

**Figure 16:**
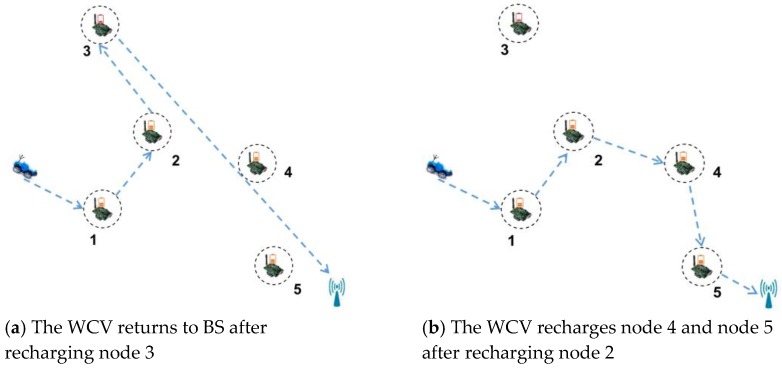
Greedy mobile scheme based on maximum recharging benefit.

However, GMS-MRB has some limitations. On the one hand, the WCV may stay away from BS in pursuit of high recharging efficiency only and it needs to return to BS in advance for replenishing its energy, which results in a greater energy consumption for moving. On the other hand, the relatively simple selection criterion ignores the possibility of recharging more other nodes. For example, in Figure 16a, it is assumed that the nodes’ ID in *Ф* are 1, 2, 3, 4, 5 at this moment, and the moving path of WCV is the blue arrow. According to GMS-MRB, the WCV is assumed to firstly recharge node 1, 2 and 3 in turn. After recharging node 3, it finds that its residual energy is not enough to recharge other nodes. At this moment, it will return to BS immediately, which probably causes node 4 and node 5 die because of untimely recharging. On the contrary, after recharging node 2, if node 3 with the maximum recharging benefit is not selected, there is a great chance for the WCV to recharge node 4 and node 5, and also, it may return to BS successfully, as shown in Figure 16b. At this time, only node 3 in the network may die, which is better than the situation shown in Figure 16a. 

Thus, we propose a wireless recharging scheme based on limited battery capacity and the maximum recharging benefit. When the recharging requests are received from nodes, the WCV firstly examines its residual energy:

Case 1: If the residual energy is no less than *γ* (calculated by Equation (35)), the WCV calculates out the remaining lifetime of each node (denoted as *L_i_*) in *Ф* by the following Equation, where $E_{i}^{R}(t)$ represents the current residual energy of node *i*:

(32) $$L_{i}=E_{i}^{R}\left(t\right)/P_{i}$$

Then, the shortest waiting time for all the other nodes is calculated in advance. For example, if node *i* is selected as the next node for recharging, the waiting time of node *i*′ (denoted as *W_ii_*_′_) is:

(33) $$W_{ii\prime }=T_{\mathrm{WCV}\;\mathrm{to}\;i}+\left(C_{b}-\left(P_{i}\times T_{j}-P_{i}\times T_{\mathrm{WCV}\;\mathrm{to}\;i}\right)\right)/\eta +T_{i\;\mathrm{to}\;i^{\prime }}$$

*T*_WCV to *i*_ and *T_i_*_to *i*′_ represent the time taken for WCV to move from its current position to node *i* and from node *i* to node *i*′, respectively. If *L_i_*_′_ ≥ *W_ii′_*, it means that node *i*′ will not die because its remaining lifetime is longer than the time on waiting for recharging when node *i* is chosen as the next node. Furthermore, if all the other nodes in *Ф* can meet the above constraint, node *i* will be added to the candidate node set (denoted as *Ψ*) for recharging. 

If *Ψ* is not empty, the node with the maximum recharging benefit will be selected as the next node for recharging. Then, its ID is removed from *Ф*, and *Ψ* is set to empty. Otherwise, the WCV inspects each node (i.e., node *i*) in *Ф* separately and calculates the number of nodes *i*′ (denoted as *Q*) that meets *L_i_*_′_ *≥ W_ii_*_′_ when node *i* is assumed to be the next node for recharging. Then, the node with the maximal value of *Q* is regarded as the next node for recharging, which minimizes the number of dead nodes. If there is more than one node has the same largest value of *Q*, the node with the maximum recharging benefit of them is selected as the next node for recharging. At last, its ID is removed from *Ф*.

After recharging the selected node, the WCV judges again whether its residual energy is still no less than *γ*. If so, it continues recharging nodes according to the strategy proposed in case 1, until *Ф* is empty, otherwise, the scheme described in case 2 is executed.

Case 2: If the residual energy of WCV is less than *γ*, nodes in *Ф* are examined in turn to judge whether they meet Equation (34).

(34) $$E_{\mathrm{WCV}}^{R}\left(t\right)-e_{m}\times d_{\mathrm{WCV}\;\mathrm{to}\;i}-\left(C_{b}-E_{i}^{R}\left(t+T_{\mathrm{WCV}\;\mathrm{to}\;i}\right)\right)\geq e_{m}\times d_{i\;to\;BS}$$

Here, $E_{\mathrm{WCV}}^{R}\left(t\right)$ is the residual energy of WCV at the current time, and $E_{i}^{R}$ (*t* + *T*_WCV to *i*_) represents the residual energy of node *i* at the moment when the WCV arrives at it. *d_i to BS_* is defined as the Euclidean distance between node *i* and BS. Similarly, *d*_WCV to_ *_i_* is defined as the Euclidean distance between the current location of WCV and node *i*. That is to say, the WCV must firstly judge whether it has enough energy to return to BS after recharging the next node. 

Nodes that do not meet the above condition are removed from *Ф.* Then, if *Ф* is not empty, the next node for recharging is selected out according to the method described in case 1, otherwise, the WCV will no longer respond to any recharging request and it immediately returns to BS to replace its battery. The time spending on battery replacement is ignored in this paper.

From the previous analysis, it is known that, under the minimum value of *γ*, the WCV should be able to return to BS, no matter where it is. In Section 4.2, we know that the theoretical longest distance between WCV and BS is $\sqrt{{\left(M/2\right)}^{2}+{\left(L/2\right)}^{2}}$. Therefore, the value of *γ* only needs to meet the following Equation:

(35) $$\gamma \geq e_{m}\times \sqrt{{\left(M/2\right)}^{2}+{\left(L/2\right)}^{2}}$$

It is worth mentioning that, the WCV can respond to the recharging requests at any time and update the contents of *Ф* constantly. 

**Figure 17:**
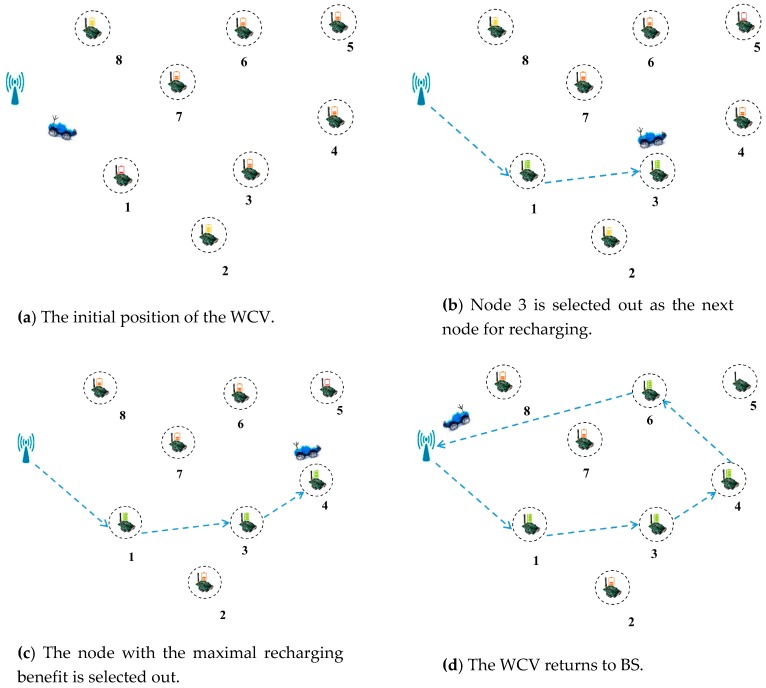
Wireless recharging scheme based on limited battery capacity and the maximum benefit.

We take Figure 17 as an example to show a specific implementation process of DCMRB. The initial position of the WCV is shown in Figure 17a and its residual energy is no less than *γ*. We assume that the nodes’ ID in *Ф* now is {1, 3, 4, 5, 6, 7}. In addition, according to Equations (32) and (33), the values of *L_i_*_′_ and *W_ii_*_′_ are calculated out respectively. It is assumed that when node 1, 3, or 7 is set as the first node for recharging, the rest of nodes will not die for the time being. Then, their IDs are added to *Ψ*. If node 1 has the maximum recharging benefit, it becomes the first node for recharging, and its ID is removed from *Ф* after it being recharged. At the same time, *Ψ* is also set to empty. After node 1 being recharged, if the residual energy of WCV is still no less than *γ*, the next node for recharging is selected out, i.e., node 3 (Figure 17b).

It is assumed that *Ψ* is empty after node 3 being recharged. Then, the node with the maximal value of *Q* is selected as the next node for recharging according to the scheme proposed in case 1. It supposes that the value of *Q* of both node 4 and node 7 are the maximum value among the remaining nodes in *Ф* at this moment. In other words, whether node 4 or node 7 is chosen as the next node for recharging, the number of dead nodes can be minimized. However, it is not possible for WCV to recharge both node 4 and node 7 at the same time. Therefore, the node with the maximal recharging benefit among them is selected out (i.e., node 4), as shown in Figure 17c.

Assuming that the residual energy of the WCV is less than *γ* after recharging node 4, the WCV needs to determine whether it has enough energy to return to BS after finishing the next recharging process on the basis of Equation (34). Without loss of generality, we assume that the WCV receives two other recharging requests from node 2 and node 8 before this judgment. So, at this time, *Ф* = {5, 6, 7, 2, 8}. Afterwards, the WCV finds that only the next node for recharging is node 6, 7, or 8, there is enough energy for it to return to BS. Thus, it selects one of these three nodes as the next node for recharging (i.e., node 6), and removes its ID from *Ф*. 

After finishing recharging node 6, WCV finds that no node meets the requirement of Equation (34). That is to say, no matter which node is recharged next, the WCV cannot return to BS. Therefore, it is no longer ready to recharge any other nodes, but immediately returns to BS, as shown in Figure 17d.

## 6. Simulation Results and Analysis

To evaluate the performance of DCMRB on multiple-MDCs based data collection strategy as well as the adaptive recharging scheme, a series of simulations are carried out with the help of Eclipse 4.5 and Matlab 8.5. Simulation results of DCMRB are also compared with some typical recharging methods, e.g., Energy Starvation Avoidance Online Charging Scheme (ESAOC), Greedy Mobile Scheme based on Maximum Recharging Benefit (GMS-MRB) and First-Come First-Served (FCFS) method. Values of the parameters in those experiments are shown in Table 4. 

### 6.1. The Minimum Number of MDCs

As mentioned in Section 4, a reasonable network partition mode will greatly affect the performance of data collection and wireless recharging. Moreover, in this paper, the number of regions is relevant to the number of MDCs. Without loss of generality, the length and width of the network are set to 400 m and 300 m respectively.

It is known from Equation (21) that, the number of MDCs is mainly affected by the value of *T_r_.* On the other hand, according to the node’s buffer size and its sensing rate, we can know that the buffer overflow will occur when its data cannot be uploaded to the MDC within 800 s. Thus, if the values of *T_d_* and *R_t_* can avoid buffer overflow, *T_r_* is calculated by the right part of Equation (15), otherwise, *T_r_* is set to 800 s.

It is not difficult to know from Table 5 that when the value of *T_d_* is constant, *T_r_* is less affected by the value of *R_t_*. However, when *R_t_* remains unchanged, *T_r_* increases significantly as the value of *T_d_* increases. Let the right parts of Equations (15) and (16) be equal, we get:

(36) $$T_{d}=\left(2-\frac{3\sqrt{3}\times g\times N}{2u\times M\times L}\times R_{t}^{2}\right)\times \frac{C}{g}-\frac{\sqrt{3}R_{t}}{v_{DGV}}$$

After calculating, it is known that the threshold value of *T_d_* is 1538 s. That is, when *T_d_* is greater than this value, in order to avoid buffer overflow, *T_r_* should not increase anymore. Therefore, in Table 5, no matter *T_d_* is 1600 s or 1700 s, the value of *T_r_* is still 800 s.

**Figure 18:**
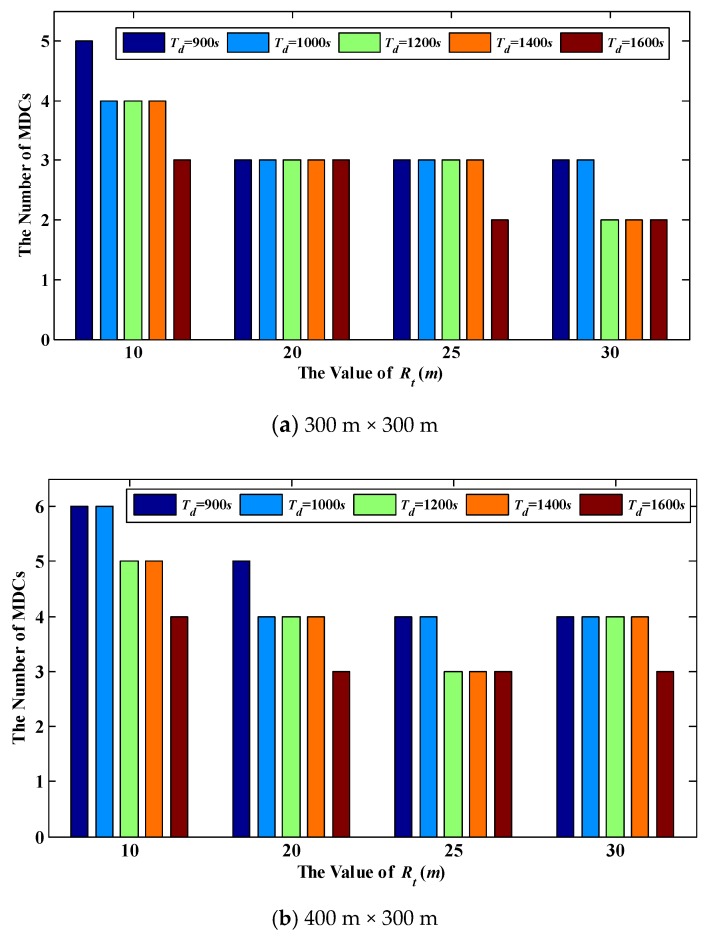

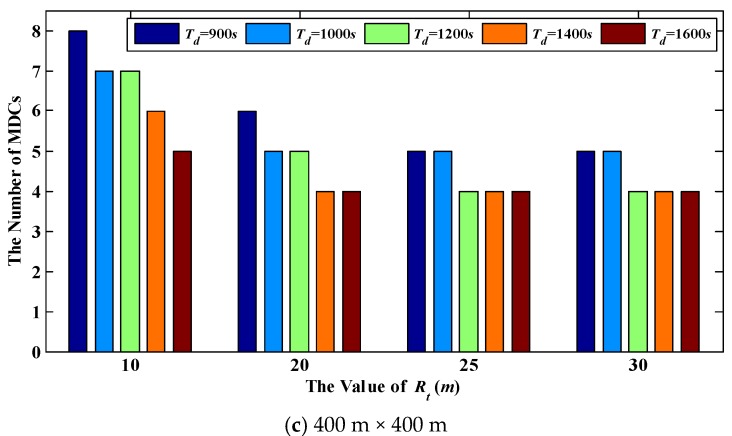
The number of MDCs in the network with different sizes.

Figure 18 show the number of MDCs in the network with different sizes. As can be seen from this figure, under the same value of *R_t_* and *T_d_*, the number of MDCs increases with the expansion of the network size. This is because once *R_t_* and *T_d_* remain unchanged, the time length of the data collection period will be fixed. Hence, the number of VTPs that one MDC can traverse in *T_r_* is also a fixed value. In this case, with the expansion of the network size, the number of VTPs increases as well, which inevitably requires more MDCs to participate in data collection.

On the other hand, under the same network size, when the value of *R_t_* or *T_d_* increases (the value of *T_d_* cannot exceed 1583 s), the number of MDCs will decrease accordingly. The reason is that the value of *T_r_* rises when *R_t_* or *T_d_* increases, and more VTPs can be traversed by WCV in *T_r_*. As a result, the demand for MDCs is reduced in the network. However, as mentioned before, in order to avoid buffer overflow, the value of *T_r_* cannot be unrestricted increased. In other words, the number of MDCs has a lower limit value. For example, in a 400 m × 400 m network, when *R_t_* is set to 25 m, the minimum number of MDCs needed is 4, as shown in Figure 18c. In Figure 18b, we can find that in the 400 m × 300 m network, the value of *R_t_* is rised from 25 m to 30 m when *T_d_* is up to 1200 s. At the same time, the number of MDCs required in this network increases from 3 to 4. It can be concluded that, although the number of VTPs decreases with the increase of *R_t_*, the Euclidean distance between two adjacent VTPs becomes longer, which prolongs the travel time of the MDC. In addition, another result of increasing the length of *R_t_* is that more nodes are contained in each RH. This results in more time needed for MDC to collect data. In this case, a MDC can only traverse less VTPs in *T_r_*, and thus more MDCs are required in the network.

### 6.2. The Amount of Data Collected before and after Speed Adjustment

**Figure 19:**
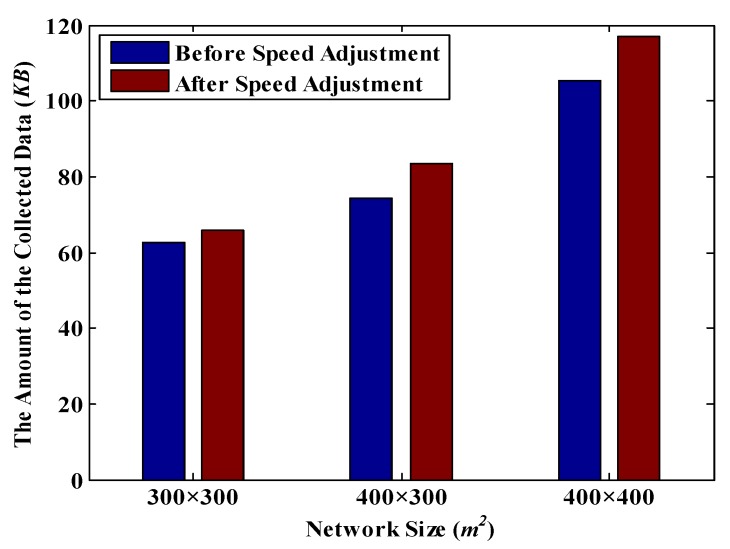
The amount of date collected before and after speed adjustment under different network sizes.

**Figure 20:**
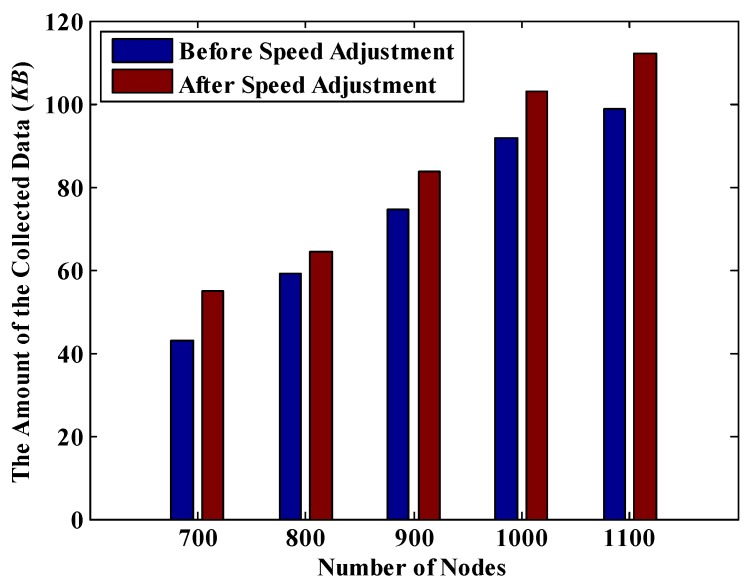
The amount of date collected before and after speed adjustment under different number of nodes.

In this section, the values of *T_d_* and *R_t_* are set to 1200 s and 30 m respectively. Thus, it is known from Table 5 that, *T_r_* = 612 s. According to Figure 19, under the same network size, the amount of collected data is increased after implementing the MDC’s speed adjustment scheme. Moreover, the larger the network size is, the greater the improvement is. For example, in a 300 m × 300 m network, the amount of the collected data is increased by 5.2%, while in a 400 m × 400 m network, the increase reaches to 11.2%. Similarly, in Figure 20, no matter how many nodes are in the network, the amount of the collected data after adjusting the speed of MDC is higher than that before adjusting. These experimental results demonstrate that after implementing the speed adjustment scheme, the packet loss rate of the whole network decreases significantly. 

### 6.3. Effect of Recharging Rate on the Performance of WRSN

The performance of WRSN is mainly affected by the following two factors:

- The Proportion of the Dead Nodes (PDN): The ratio of the number of dead nodes to the total number of nodes deployed in the network.
- The Average Recharging Delay (ARD): The average time interval from the moment when a node sending a recharging request to the moment when it being recharged.

**Figure 21:**
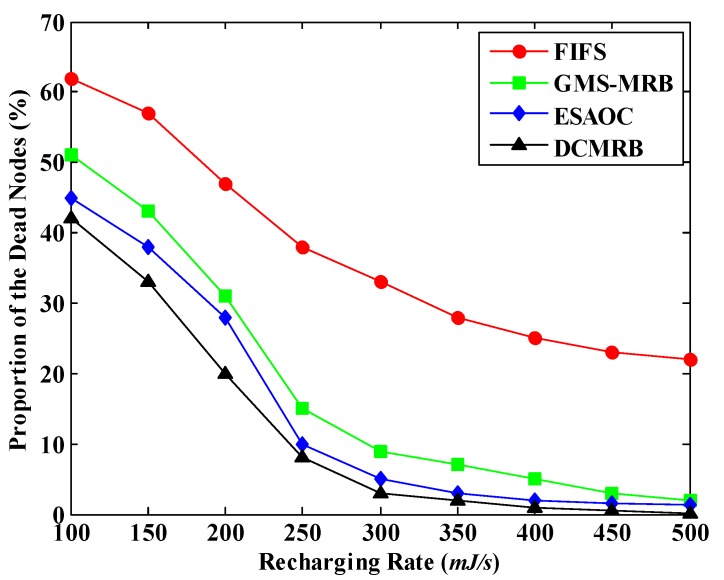
Proportion of the dead nodes under different recharging rates.

Without loss of generality, the values of *T_d_* and *R_t_* are set to 1200 s and 20 m respectively in the following sections. Figure 21 shows the simulation result of the PDN under different recharging rates. We can observe that with the increase of *η*, the PDN of all the four methods drops significantly. In this case, more and more nodes can be recharged in the same time period, which prevents the node from dying due to energy exhaustion.

However, it should be pointed out that the PDN of FIFS is always the highest among the four methods. Even when *η* = 500 mJ/s, its value is still higher than 20%. This is because FIFS simply follows the principle of “first-come, first-served” to recharge the requesting node. This easily causes the WCV moving back and forth between nodes farther away, which not only consumes more energy, but also increases the waiting time of nodes to be recharged. Therefore, the number of nodes that can be recharged in FIFS is small.

On the other hand, in GMS-MRB, the recharging benefit is regarded as the only criterion to select the next recharging node. This may make the WCV keep away from the BS due to the pursuit of high recharging efficiency. In this case, the WCV consumes much energy on moving, and it may have to return to BS in advance, as shown in Figure 16a. In summary, the PDN of GMS-MRB is also higher than that of DCMRB.

**Figure 22:**
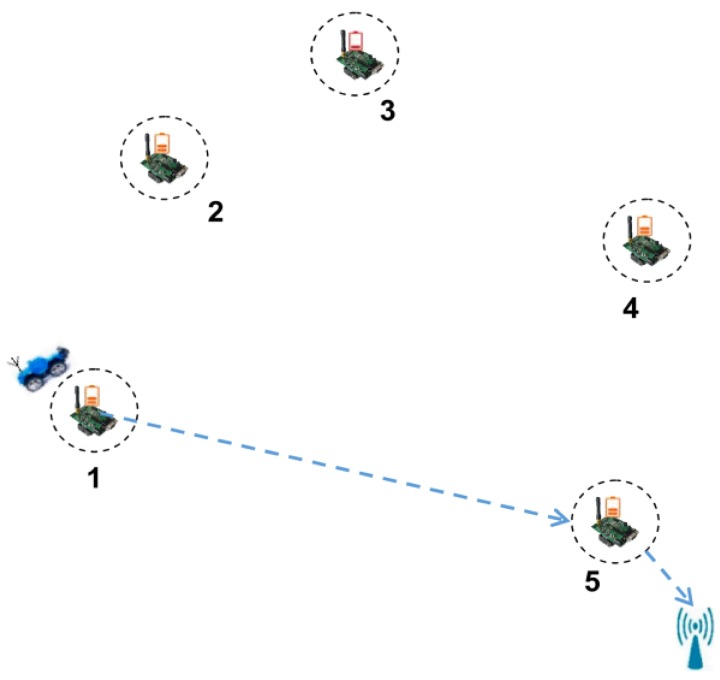
An example about the next recharging node selection strategy of ESAOC.

Although the PDN of ESAOC is close to that of DCMRB, it is still slightly higher. The main reason is that in ESAOC, when the WCV finishes recharging a node, it judges whether or not it has enough energy to return to BS, which undoubtedly increases its computation overhead. It is even more so in a WRSN where nodes are densely deployed. In addition, due to falling into local optimum, the number of nodes to be served by WCV tends to be reduced. For example, in Figure 22, five nodes have sent the recharging requests. It is assumed that the WCV is now at node 1 and has just finished recharging it. Subsequently, node 2 is selected as the next node for recharging according to the strategy of ESAOC. However, after judgment, it finds that if the WCV moves to node 2 and recharges it, it doesn’t have enough power to return to BS. Hence, the WCV reselects node 3, which is closest to node 2, as the next recharging target. Unfortunately, by judging again, the WCV finds that it still cannot return to BS if recharging node 3. Therefore, the WCV has no choice but to give up recharging all the other nodes and return to BS. Since node 5 is very close to BS, it is possible for the WCV to recharge it and then return to BS. However, ESAOC fails to do this. In contrast, DCMRB has established a recharging request queue and it has also forecasted the situation after recharging each node in this queue, so that the most suitable node can be selected out for each recharging.

**Figure 23:**
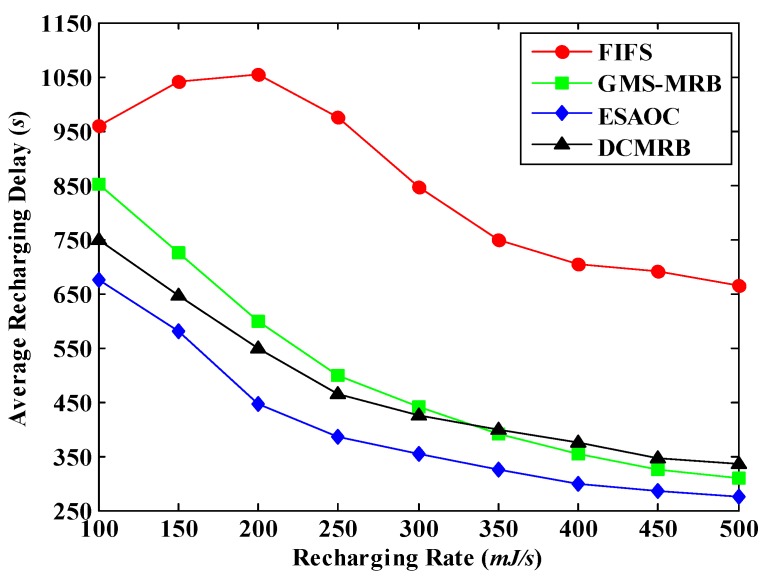
Average recharging delay under different recharging rates.

As shown in Figure 23, the ARD of all the four methods shows a decreasing trend with the increase of the recharging rate. However, in FIFS, when the recharging rate rises from 0.1 J/s to 0.2 J/s, the average recharging delay increases slightly. This is because FIFS only relies on the sequence of the recharging requests issued by nodes for recharging, and it fails to plan a reasonable moving path for the WCV, which may cause the WCV moving back and forth between nodes farther apart. When the recharging rate increases, the WCV can recharge more nodes. So, the movement situation mentioned above may be more frequent, resulting in a certain rise of the ARD. However, when the recharging rate further increases (e.g., *η* = 0.2 J/s), the ARD of FIFS keeps falling due to the improvement of the recharging performance (but it is still the highest of the four methods).

When *η* is small, the average recharging delay of GMS-MRB is higher than that of ESAOC and DCMRB. In this case, the large value of PDN leads to the decrease of node’s density in the network, which increases the average distance between two consecutive rechargeable nodes. So, the recharging delay is higher. When *η* is large, it is most possible to select the node that closest to WCV as the next recharging node. Although this node may not be the best option, it can greatly reduce the recharging delay. Therefore, it can be seen from Figure 23 that the average recharging delay of GMS-MRB is even lower than that of DCMRB when *η* > 0.32 J/s.

In order to reduce the recharging time, ESAOC preferentially selects nodes with more residual energy to recharge so as to respond more requests. Thus, the average recharging delay of ESAOC is always the lowest of the four methods. However, it is worth mentioning that this may overlook the urgency of recharging, making some nodes with low residual energy more likely to die.

Different from the other three methods, the path length and the number of nodes that can be served are taken into account in DCMRB. On this basis, it always selects the node with the maximum benefit as the next recharging target, and the WCV recharges each node to the full power (*C_b_*) every time. In summary, its average recharging delay is basically the same as that of ESAOC and GMS-MRB, but much lower than FIFS.

### 6.4. Effect of the Moving Speed of WCV on the Performance of WRSN

**Figure 24:**
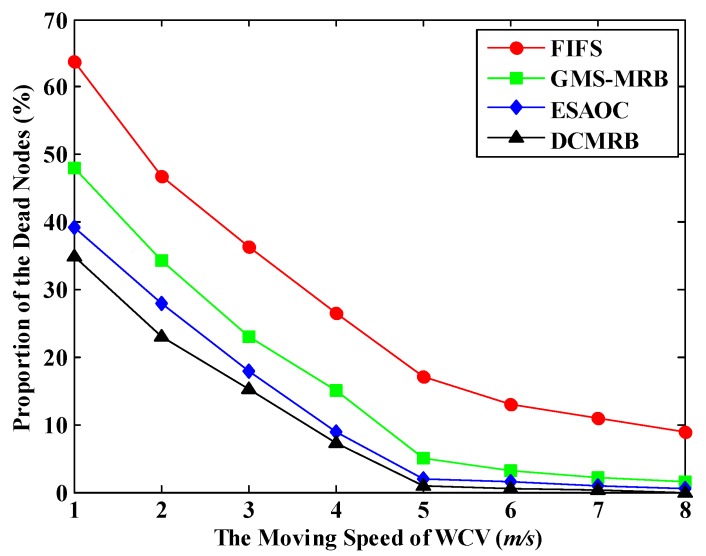
Proportion of the dead nodes under different moving speeds.

Figure 24 shows the PDN of the four methods under different moving speeds of WCV. It is obvious that the largest range of change appears in FIFS. When the WCV’s moving speed gradually varies from 1 m/s to 8 m/s, the value of PDN drops by about 54%. This is still because FIFS only recharges nodes by sequence based on their request times, easily resulting in excessive time consumption on moving. After increasing the moving speed of WCV, the waiting time of nodes for recharging decreases and the value of PDN goes down. However, since the movement mode of WCV does not change, it still wastes much energy on moving. Therefore, under the same value of the moving speed, the PDN of FIFS is still the highest among the four methods.

Although the PDN of GMS-MRB is lower than that of FIFS, it is always higher than DCMRB and ESAOC. The reason is that the WCV may stay away from BS in pursuit of high recharging efficiency only and it also needs to return to BS in advance for replenishing energy, which causes a greater reduction on the number of nodes being recharged. Even if the moving speed increases, it is impossible to fully compensate for the time and energy costs due to long-distance movement. 

Obviously, the PDN of DCMRB or ESAOC is relatively lower, especially when the speed is greater than or equal to 5 m/s. Unlike the uniform recharging threshold used in ESAOC, DCMRB adopts a more flexible mechanism to adapt different energy consumption rates of nodes. So, the PDN of DCMRB is the lowest among the four methods.

**Figure 25:**
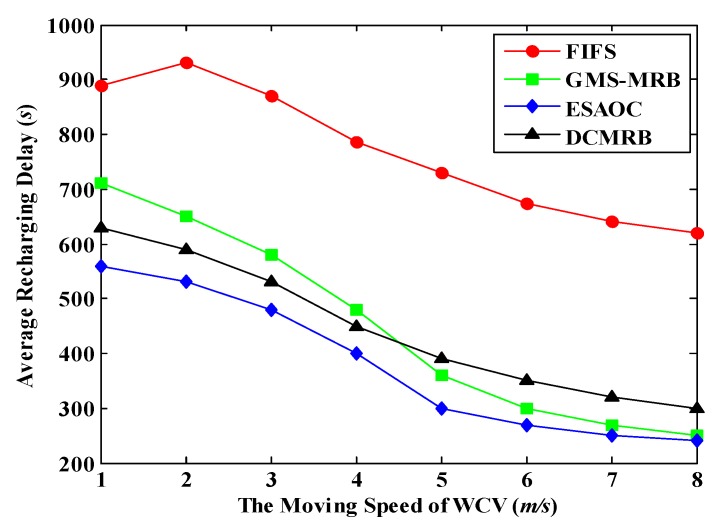
Average recharging delay under different moving speeds.

Figure 25 shows the average recharging delay under different moving speeds of WCV. As expected, in all the four methods, the value of ARD decreases with the increase of *v*_WCV_. However, in FIFS, when *v*_WCV_ increases from 1 m/s to 2 m/s, the average recharging delay goes up slightly. The reason of this is similar to the case in Figure 23.

From Figure 25, we can also see that the average recharging delay in ESAOC is still the lowest among the four methods. The experimental results of DCMRB are close to ESAOC, which is similar to the conclusion of Figure 23. 

It is worth mentioning that the average recharging delay of GMS-MRB is higher than that of DCMRB and ESAOC when the WCV moves at a slower speed. However, when *v*_WCV_ ≥ 5 m/s, the average recharging delay of GMS-MRB is slightly lower than that of DCMRB. In this case, there are few dead nodes in the network and the WCV can easily find a rechargeable node that is close to its current location, which effectively shortens the waiting time of nodes for recharging. However, DCMRB aims to find “the most suitable node for recharging”, so its path length may be longer than that of GMS-MRB, resulting in a higher latency (but the number of dead nodes in DCMRB is the lowest).

### 6.5. Effect of the Threshold of Recharging Request on the Performance of WRSN

**Figure 26:**
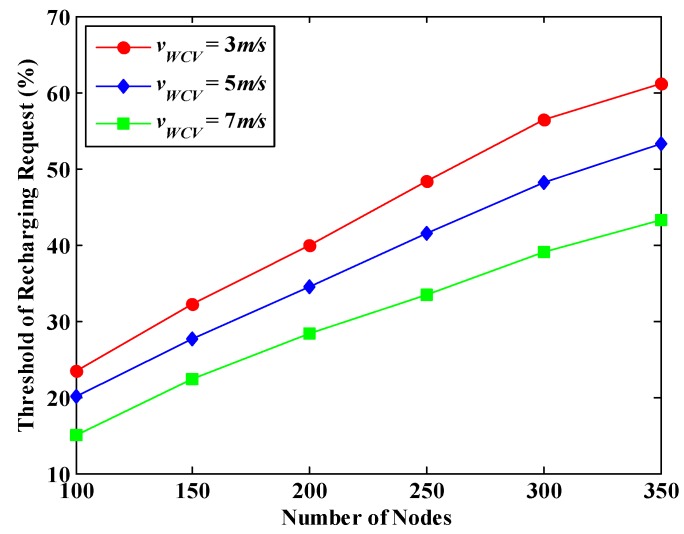
The average value of TRR under different number of nodes and different moving speeds.

Figure 26 shows the energy Threshold of Recharging Request (TRR) under different number of nodes and different moving speeds in one region of DCMRB. As described in Section 5.1, an adaptive TRR is set for each node in our algorithm. For ease of analysis, in Figure 26, the algorithm performance is measured by the average value of TRR. It is not difficult to see that, under the same value of *v*_WCV_, this average threshold goes up with the increase of the number of nodes in the network, and almost a linear growth. The reason is that the waiting time of nodes for recharging increases as the number of nodes increases, according to Equation (31). Moreover, when the number of nodes in one region is unchanged, the faster the WCV moves, the more nodes it serves, and the lower the average value of TRR is.

**Figure 27:**
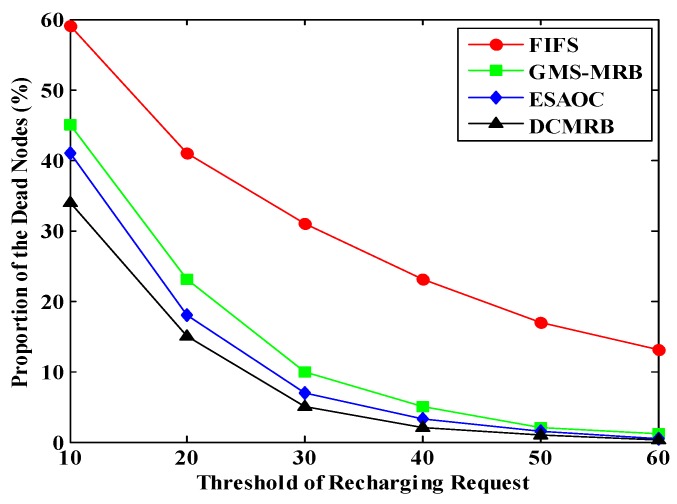
Proportion of the dead nodes under different recharging thresholds.

The value of PDN under different TRRs in the four methods is shown in Figure 27.

As the recharging threshold increases, the PDN in all the four methods decreases. Obviously, higher recharging threshold means that nodes would send the requests more frequently. Thus, the WCV can optimize the moving path more efficiently. On the other hand, when the value of TRR is raised, the WCV can complete each recharging task in a shorter time, so more nodes would be recharged and the PDN is further reduced. Among the four methods, the performance of DCMRB is the best.

### 6.6. Effect of the Recharging Rate on the Amount of the Collected Data

**Figure 28:**
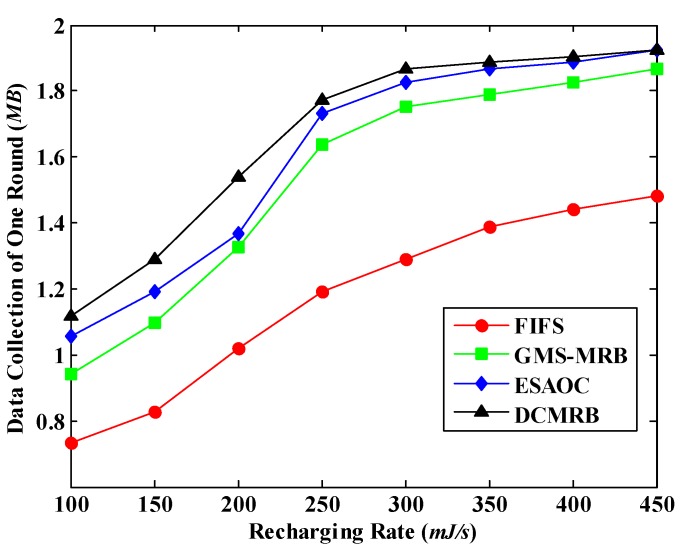
The amount of the collected data under different recharging rates.

At last, we comprehensively consider the implementation effects of WCV and MDC to verify the effect of the recharging rate on the amount of the collected data. The simulation results are shown in Figure 28. It is obvious that, with the increase of *η*, the amount of data collected in these four methods increases significantly. Combined with Figure 21, we can see that in all the four methods, PDN drops rapidly when *η* rises. That is to say, more and more active nodes exist in the network. Thus, the amount of data collected by MDC increases. In addition, when *η* ≥ 300 mJ/s, the PDN of GMS-MRB, ESAOC and DCMRB are almost unchanged any more, so is the amount of the collected data, which is also confirmed by Figure 28.

Moreover, Figure 28 also illustrates that the amount of collected data goes up dramatically when the recharging rate increases from 200 mJ/s to 250 mJ/s in ESAOC. From Figure 21, it is known that during this period, the PDN of ESAOC drops sharply from 28% to 12%. That is to say, the proportion of the surviving nodes is largely increased. Therefore, the amount of the collected data rapidly rises in the period.

## 7. Conclusions

In order to prolong the lifetime of the self-organized Wireless Sensor Networks, both a distributed data collection method and a high efficient energy supplement strategy are described in this paper. Through the virtual scan line based network partition method and the moving speed adjustment scheme, the efficiency of data collection is improved to a certain extent. Moreover, with the help of the adaptive recharging scheme based on maximum benefit, the energy is reasonably supplemented to the most needed nodes, that indirectly ensures the balance of energy consumption.

However, due to the slow moving speed and the low recharging rate of the WCV, it may not recharge all nodes timely when a mass of nodes send recharging requests during a short time. Thus, how to effectively meet the frequent recharging and data uploading requirements of the nodes in a large scale network in real time is one of our future research points. In addition, the one-to-more recharging mode may also be considered in our future work. That is, within a certain range, the WCV can recharge two or more nodes simultaneously, which can further increase the recharging efficiency.

## Figures and Tables

**Table 1:** Definitions of parameters about the network.

Parameter	Symbol	Unit
Network size	*M* × *L*	m^2^
Number of nodes	*N*	-
Sensing radius of node	*R_s_*	m
Communication radius of node	*R_t_*	m
Number of regions	*k*	-
Number of VTPs	*m*	-

**Table 2:** Definitions of parameters in multi-MDCs-based data collection.

Parameter	Symbol	Unit
Maximum time delay of packet uploading	*T_d_*	s
Speed of MDC	*v*_MDC_	m/s
The *i*th regular hexagons (*i* = 1, 2, …, *m*)	*RH_i_*	-
Number of nodes in *RH_i_*	*Num (RH_i_)*	-
Time for the MDC to stay at each VTP	*t*_s_	s
Sensing rate of node	*g*	bit/s
Data uploading rate of node	*u*	bit/s
A round of data gathering time of MDC	*T_r_*	s
Buffer size of node	*C*	bit
Number of VTPs in the *i*th region (*i* = 1, 2, …, *k*)	$Num_{i}^{\mathrm{VTP}}$	-
Path length of MDC in the *i*th region (*i* = 1, 2, …, *k*)	*D_i_*	m
Virtual traverse points	VTP	-
Base station	*BS*	-
Maximum time delay of packet transmission	$T_{d}^{\max}$	s
Maximum number of VTPs that MDC can traverse during *T_r_*	*m*′	-
Euclidean distance between BS and the first VTP	*d_BS to RHi_*	m
Minimum number of MDCs required in the network	*k*	-
Euclidean distance between two adjacent VTPs in the *i*th region (*i* = 1, 2, …, *k*)	*D (j* − 1, *j*)	m
Unit data collection period of MDC in the *i*th region (*i* = 1, 2, …, *k*)	*T* (*j* − 1, *j*)	s
Speed of MDC from *VTP_j_*_−1_ to *VTP_j_* after adjustment in the *i*th region (*i* = 1, 2, …, *k*)	*v*_MDC_ (*j* − 1, *j*)	m/s

**Table 3:** Definitions of parameters about the adaptive recharging scheme.

Symbol	Definition	Unit
*E_elec_*	Energy consumption of the sending and receiving circuit	nJ/bit
*ε_fs_*	Energy consumption of the amplifier in free-space model	pJ × (b/m^2^)^−1^
*P_i_*	Energy consumption rate of node *i*	J/s
*d_i_*_to VTP_	Single-hop transmission distance of node *i*	m
*e_s_*	Energy consumption rate of sensing	J/s
*N_j_*	Number of nodes in the *j*th region	-
*C_b_*	Battery capacity of sensor node	J
*C_h_*	Battery Capacity of WCV	J
*e_m_*	Energy consumption of WCV on travelling one meter	J/m
*η*	Wireless recharging rate	J/s
*v*_WCV_	Speed of WCV	m/s
*γ*	Residual energy threshold of WCV	J
*Ф*	Recharging request queue	-
*Ψ*	Queue of nodes waiting for recharging	-

**Table 4:** Parameter values.

Parameter	Symbol	Value
Network size	*M* × *L*	300 m × 300 m–400 m × 400 m
Number of nodes	*N*	923
Length of the sensing radius	*R_s_*	10 m
Length of the communication radius	*R_t_*	10 m–30 m
Speed of MDC	*v*_MDC_	5 m/s
Speed of WCV	*v*_WCV_	5 m/s
Buffer size of one node	*C*	12.7 KB
Data collection rate of one node	*g*	130 bps
Data uploading rate of one node	*u*	100 kbps
Maximum battery capacity of one node	*C_b_*	5 J
Maximum battery capacity of the WCV	*C_h_*	1 KJ
Energy consumption of the WCV on travelling one meter	*e_m_*	0.2 J
Wireless recharging rate	*η*	0.1 J/s–0.5 J/s
Energy consumption of the sending and receiving circuit	*E_elec_*	50 nJ/bit
Energy consumption of the amplifier in free-space model	*ε_fs_*	10 pJ·(b/m^2^)^−1^
Maximum delay for packet uploading	*T_d_*	900 s–1700 s

**Table 5:** The value of *T_r_* under different values of *T_d_* and *R_t_*.

		*R_t_*	10 m	20 m	25 m	30 m
	*T_r_*					
*T_d_*						
900 s			452 s	455 s	458 s	460 s
1000 s			502 s	506 s	508 s	511 s
1200 s			602 s	606 s	609 s	612 s
1400 s			702 s	707 s	710 s	713 s
1500 s			752 s	757 s	760 s	764 s
1600 s			800 s	800 s	800 s	800 s
1700 s			800 s	800 s	800 s	800 s
